# TRKB tyrosine kinase receptor is a potential therapeutic target for poorly differentiated oral squamous cell carcinoma

**DOI:** 10.18632/oncotarget.25396

**Published:** 2018-05-18

**Authors:** Kazumasa Moriwaki, Yusuke Ayani, Hiroko Kuwabara, Tetsuya Terada, Ryo Kawata, Michio Asahi

**Affiliations:** ^1^ Department of Pharmacology, Faculty of Medicine, Osaka Medical College, Takatsuki, Osaka 569-8686, Japan; ^2^ Department of Otolaryngology-Head and Neck Surgery, Faculty of Medicine, Osaka Medical College, Osaka 569-8686, Japan; ^3^ Department of Pathology, Faculty of Medicine, Osaka Medical College, Osaka 569-8686, Japan

**Keywords:** TRKB, oral squamous cell carcinoma, tumor differentiation, prognostic factors, anti-cancer drug

## Abstract

It has been reported that one of the neurotrophin receptors, tropomyosin receptor kinase B (TRKB), is frequently overexpressed in various tumor tissues including oral squamous cell carcinoma (OSCC), and that its upregulation promotes tumor progression in human cancers. However, the correlation between TRKB overexpression and clinicopathological characteristics is not fully elucidated. Here, we present the correlation between the expression levels of TRKB and/or its secreted ligand, brain-derived neurotrophic factor (BDNF), and clinicopathological characteristics, especially regarding tumor differentiation, tissue invasion, and disease-free survival in patients with OSCC. The results obtained through immunohistochemical analysis of human OSCC tumor specimens showed that the expression levels of TRKB and/or BDNF, were significantly higher in moderately and poorly differentiated OSCC (MD/PD-OSCC) tumor cells than in well differentiated cells (WD-OSCC). Moreover, the OSCC tumors highly expressing TRKB and/or BDNF exhibited promotion in tissue invasion and reduction in disease-free survival in the patients. In an orthotopic transplantation mouse model of human OSCC cell lines, administration of a TRKB-specific inhibitor significantly suppressed the tumor growth and invasion in PD-OSCC-derived tumor cells, but not in WD-OSCC-derived tumor cells. Moreover, the TRKB inhibitor selectively blocked BDNF-induced tumor cell proliferation and migration accompanied with the suppression of TRKB phosphorylation in PD-OSCC but not in WD-OSCC *in vitro*. Taken together, these data suggest that the BDNF/TRKB signaling pathway may regulate tumor progression in poorly differentiated OSCC. Expression levels of signal molecules may be an accurate prognosis marker for tumor aggressiveness, and the molecules may be an attractive target for new OSCC therapies.

## INTRODUCTION

Oral squamous cell carcinoma (OSCC) is the most common malignancy of the head and neck, and its incidence has increased in recent years, especially in younger patients [1, 2]. An estimated 300,400 new cases and 145,400 deaths from oral cavity cancer occurred in 2012 worldwide [3]. Despite advances in treatments such as surgery, radiation, chemotherapy, and targeted therapy, the recurrence and survival of patients with OSCC has not significantly improved in the last decade [4]. The poor survival benefit is due to metastasis and its poor prognosis. Metastases are found as recurrent in approximately 20–30% of patients with OSCC within 2–3 years of surgical treatment [5, 6]. Cisplatin is used in postoperative adjuvant chemotherapy or the treatment of unresectable malignancies and metastatic lesions, and shows improvement of quality of life and lifespan. However, its severe or life-threatening toxicity remains a serious problem, and it shows no significant improvement in terms of complete recovery [7]. Therefore, there is a need to develop new therapeutic targets and cancer treatment methods.

In general, tumor size, differentiation, perineural invasion, vascular invasion, and nodal metastasis are predictors of recurrence and survival in patients with OSCC. Especially, the differentiation stage of tumor is a central aspect in the histopathological classification of OSCC. The differentiation stage is strongly associated with tumor behavior, including tissue invasion and metastasis, and generally, poorly differentiated tumors are more malignant than well differentiated ones. Numerous studies show that poorly differentiated tumors exhibit aggressive local invasion and metastasis, leading to recurrence. However, despite the availability and importance of the tumor's differentiation stage in the classification and malignant feature of OSCC, tumor differentiation grade is not utilized toward OSCC treatment, especially drug treatment. It is used for the classification of OSCC only, owing to the poor understanding of the molecular profiling in patients with OSCC regarding the correlation between tumor differentiation and OSCC progression. Therefore, improved understanding of the molecular classification of OSCC in tumor differentiation is of utmost clinical and fundamental importance for diagnosis and therapy.

Progress in understanding the fundamental mechanisms of tumor progression and metastasis has yielded promising targets for therapeutic applications in various cancers. The epidermal growth factor receptor (EGFR/ErbB1/HER1) seems to be a particularly promising target for therapy of OSCC. Elevated expression of EGFR is widely found in OSCC, and its overexpression is associated with worse prognosis [7–12]. To date, a monoclonal antibody targeting EGFR, cetuximab, is used for clinically approved targeted therapy of OSCC. Although cetuximab is effective in locoregional control and disease-free survival in patients with OSCC, it shows no significant improvement in terms of complete recovery [10, 13, 14]. Furthermore, the resistance to cetuximab has been reported [15, 16]. To improve complete recovery in patients with OSCC, metastasis-related molecules might need to be considered as targets for the treatment.

The tropomyosin receptor kinase (TRK) family is composed of tyrosine kinase receptors including TRKA, TRKB, and TRKC, encoded by *NTRK1, NTRK2,* and *NTRK3* genes, respectively. Although it is well-known that TRK proteins play a wide variety of roles in neuronal function during developmental, physiological, and disease processes, they were initially identified in cancer [17–19]. In central and peripheral nervous systems, TRK proteins serve as high-affinity receptors for the nerve growth factor, a type of neurotrophins (brain-derived neurotrophic factor, BDNF; neurotrophin-3, NT-3; and neurotrophin-4, NT-4), and play a critical role in regulating neuronal cell survival, neurite growth, cell migration, spine and dendritic growth, and synapse formation [20–23]. Overexpression of TRK proteins and *NTRK* gene fusion were found in many types of cancer [21, 24–28]. Especially, TRKB is well investigated in many types of cancer. TRKB promotes tumor cell proliferation through the activation of the RAS/MAPK, the PI3K/PDK1/AKT, and the PLCγ pathways [29] and induces anoikis suppression and epithelial-mesenchymal transition through the induction of Twist and Snail [30–33]. It has also been established that TRKB promotes tumor metastasis in some types of tumors, such as lung adenocarcinoma [34, 35], breast cancer, and neuroblastoma [36], using tumor transplantation mouse models. Overexpression of TRKB and its specific ligand, BDNF, in OSCC, was also reported by several research groups [22, 32, 37, 38]. However, regardless of these accumulating findings, the correlation between TRKB overexpression and clinicopathological characteristics in patients with OSCC is not fully elucidated.

In this study, we used the human OSCC tissues to examine the correlations between TRKB/BDNF expression, tumor differentiation, and clinicopathologic features in patients with OSCC. We also used two different types of human OSCC cell lines (well differentiated and poorly differentiated) to study the effect of a TRKB-specific inhibitor for tumor therapy in tumor cell-transplanted mouse models and cell culture systems. We describe a new correlation between TRKB/BDNF overexpression and OSCC tumor differentiation, and propose that TRKB is a potential therapeutic target for OSCC, especially for poorly differentiated OSCC.

## RESULTS

### Clinicopathologic features in patients with OSCC

The preferential site of the OSCC was the tongue (31/44 cases, 70.5%), followed by the mouth floor (5/44 cases, 11.4%), gingiva (5/44 cases, 11.4%), buccal mucosa (2/44 cases, 4.5%), and hard palate (1/44 case, 2.3%). All 44 patients were classified as either stage I (25/44 cases, 56.8%), stage II (15/44 cases, 34.1%), stage III (2/44 cases, 4.55%), or stage IV (2/44 cases, 4.55%). According to the Tumor-Node-Metastasis (TNM) clinical classification, 25 cases were classified as T1 (56.8%) and 19 as T2 (43.2%). Tumor grading showed that 25 cases were well differentiated (56.8%), 12 cases were moderately differentiated (27.3%), and 7 cases were poorly differentiated (15.9%).

### Correlation between the expression levels of TRKB, BDNF, or both, and clinicopathological features in patients with OSCC

**Figure 1:**
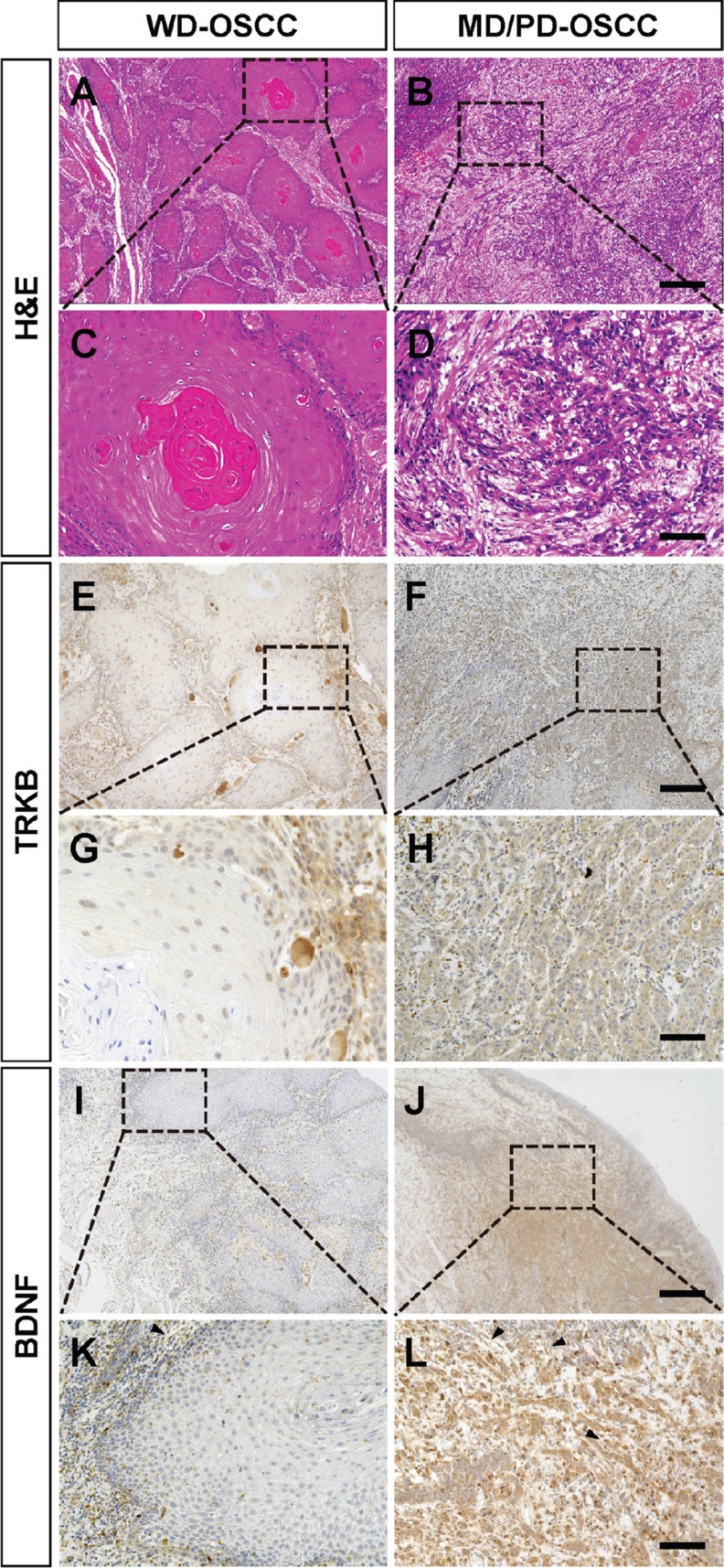
Higher expressions of TRKB and BDNF in moderately or poorly differentiated human OSCC (**A**–**D**) Representative H&E staining images of the tumor tissues from patients with OSCC. (**E**–**L**) Immunohistochemical detection of TRKB (E–H) and BDNF (I–L) in the tumor tissues. Representative images of well differentiated (WD) and moderately/poorly differentiated (MD/PD) OSCC tumors are shown in the left and right panels, respectively. Bars represent 500 μm (A, B, E, F, I, and J) and 100 μm (C, D, G, H, K, and L).

Despite the accumulating understanding of the basic molecular functions of TRKB, the correlation between the expression levels of TRKB, BDNF, or both, and the clinical significance in patients with OSCC is not well understood. First, we analyzed the expression levels of TRKB and its specific ligand, BDNF, in OSCCs from 44 patients who had not received any previous treatment, by immunohistochemistry. In most cases, the expression levels of TRKB and BDNF were both low (TRKB^low^/BDNF^low^) in WD-OSCC tumor cells, whereas they were both higher (TRKB^high^/BDNF^high^) in MD and PD-OSCC tumor cells (Figure 1). The expression level of BDNF was negative or very low in the normal-appearing oral mucosae adjacent to OSCC, whereas TRKB was weakly expressed in the stratum consisting of proliferating cuboidal cells (Supplementary Figure 1). In OSCC tumor lesion, TRKB and BDNF were highly expressed in infiltrated immune cells and tumor-associated vessels as well as in tumor cells. (Supplementary Figure 2). To clarify whether these data are significant or not, we statistically analyzed the correlation between the expression levels of TRKB, BDNF, or both, and the clinicopathological significances in all 44 patients with OSCC (Tables 1 and 2). High expression level of BDNF was found in 64.0% of TRKB-positive OSCC (16/25). There were no significant correlations between TRKB expression and clinicopathological characteristics, such as gender, T classification, or stage. Similar trends were observed for BDNF. On the other hand, TRKB expression level was significantly correlated with lymphovascular invasion (*P* = 0.049), whereas BDNF expression level was significantly correlated with muscle invasion (*P* = 0.036) and, to a moderate extent, associated with lymphovascular invasion (*P* = 0.081) and neuroinvasion (*P* = 0.062). TRKB^high^/BDNF^high^ also tended to be associated with lymphovascular invasion (*P* = 0.051) and muscle invasion (*P* = 0.095) (Table 3). Importantly, the high expression levels of TRKB, BDNF, or both were observed in 100% (19/19), 73.7% (14/19), and 73.7% (14/19) of MD/PD-OSCC, respectively, contrasting with the 24.0% (6/25), 16.0% (4/25), and 8.0% (2/25) detected in WD-OSCC (*P* < 0.001). Additionally, we analyzed the correlations between tumor differentiation and other clinicopathologic significances in Table 4. There was a significant correlation between tumor differentiation and lymphovascular invasion (*P* = 0.011). To a lower extent, tumor differentiation was also associated with neuroinvasion (*P* = 0.073).

**Table 1:** Correlation between TRKB expression level and the clinicopathological characteristics of the 44 OSCC patients

Variables		TRKB		*P* value
		Low	High	
Gender	M	10	13	0.97
	F	9	12	
T classification	T1	10	15	0.63
	T2	9	10	
Stage	I	10	15	0.63
	II, III, IV	9	10	
Nodal metastasis	–	18	22	0.42
	+	1	3	
Lymphovascular invasion	–	19	20	0.049^*^
	+	0	5	
Vascular invasion	–	17	22	0.63
	+	2	3	
Neuroinvasion	–	19	22	0.17
	+	0	3	
Muscle invasion	–	9	9	0.45
	+	10	16	
Differentiation	Well	19	6	<0.001^***^
	Mod/Por	0	19	
BDNF expression	Low	17	9	<0.001^***^
	High	2	16	

Statistical analyses were performed using Pearson's χ^2^ test or Fisher's exact test. ^*^P < 0.05 and ^***^P < 0.001. M, male; F, female; Well, well differentiated OSCC; Mod/Por, moderately or poorly differentiated OSCC.

**Table 2:** Correlation between BDNF expression level and the clinicopathological characteristics of the 44 OSCC patients

Variables		BDNF		*P* value
		Low	High	
Gender	M	13	10	0.72
	F	13	8	
T classification	T1	14	11	0.63
	T2	12	7	
Stage	I	14	11	0.63
	II, III, IV	12	7	
Nodal metastasis	–	25	15	0.18
	+	1	3	
Lymphovascular invasion	–	25	14	0.081
	+	1	4	
Vascular invasion	–	23	16	0.67
	+	3	2	
Neuroinvasion	–	26	15	0.062
	+	0	3	
Muscle invasion	–	14	4	0.036^*^
	+	12	14	
Differentiation	Well	21	4	<0.001^***^
	Mod/Por	5	14	
TRKB expression	Low	17	2	<0.001^***^
	High	9	16	

Statistical analyses were performed using Pearson's χ^2^ test or Fisher's exact test. ^*^P < 0.05 and ^***^P < 0.001. M, male; F, female; Well, well differentiated OSCC; Mod/Por, moderately or poorly differentiated OSCC.

**Table 3:** Correlation between TRKB/BDNF co-overexpression levels and the clinicopathological characteristics of the 44 OSCC patients

Variables		TRKB/BDNF		*P* value
		Low	High	
Gender	M	15	8	0.82
	F	13	8	
T classification	T1	15	10	0.57
	T2	13	6	
Stage	I	16	9	0.95
	II, III, IV	12	7	
Nodal metastasis	–	26	14	0.46
	+	2	2	
Lymphovascular invasion	–	27	12	0.051
	+	1	4	
Vascular invasion	–	25	14	0.61
	+	3	2	
Neuroinvasion	–	26	13	0.25
	+	2	3	
Muscle invasion	–	14	4	0.095
	+	14	12	
Differentiation	Well	23	2	<0.001^***^
	Mod/Por	5	14	

Statistical analyses were performed using Pearson's χ^2^ test or Fisher's exact test. ^*^P < 0.05 and ^***^P < 0.001. M, male; F, female; Well, well differentiated OSCC; Mod/Por, moderately or poorly differentiated OSCC.

**Table 4:** Correlation between the histological differentiation and other clinical factors for OSCC

Variables		Differentiation		*P* value
		Well	Mod/Por	
Gender	M	12	11	0.52
	F	13	8	
T classification	T1	16	9	0.27
	T2	9	10	
Stage	I	16	9	0.27
	II, III, IV	9	10	
Nodal metastasis	–	24	16	0.21
	+	1	3	
Lymphovascular invasion	–	25	14	0.011^**^
	+	0	5	
Vascular invasion	–	23	16	0.37
	+	2	3	
Neuroinvasion	–	25	16	0.073
	+	0	3	
Muscle invasion	–	12	6	0.27
	+	13	13	
TRKB expression	Low	19	0	<0.001^***^
	High	6	19	
BDNF expression	Low	21	5	<0.001^***^
	High	4	14	
TRKB/BDNF expression	–	23	5	<0.001^***^
	+	2	14	

Statistical analyses were performed using Pearson's χ^2^ test or Fisher's exact test. ^*^P < 0.05, ^**^P < 0.01, and ^***^P < 0.001. M, male; F, female; Well, well differentiated OSCC; Mod/Por, moderately or poorly differentiated OSCC.

These data show that the expression levels of TRKB, its ligand BDNF, or both, are significantly elevated in MD/PD-OSCC tumor cells and associated with OSCC tumor invasion.

### Correlation between disease-free survival and the expression levels of TRKB, BDNF, or both, as well as tumor cell differentiation, in patients with OSCC

**Figure 2:**
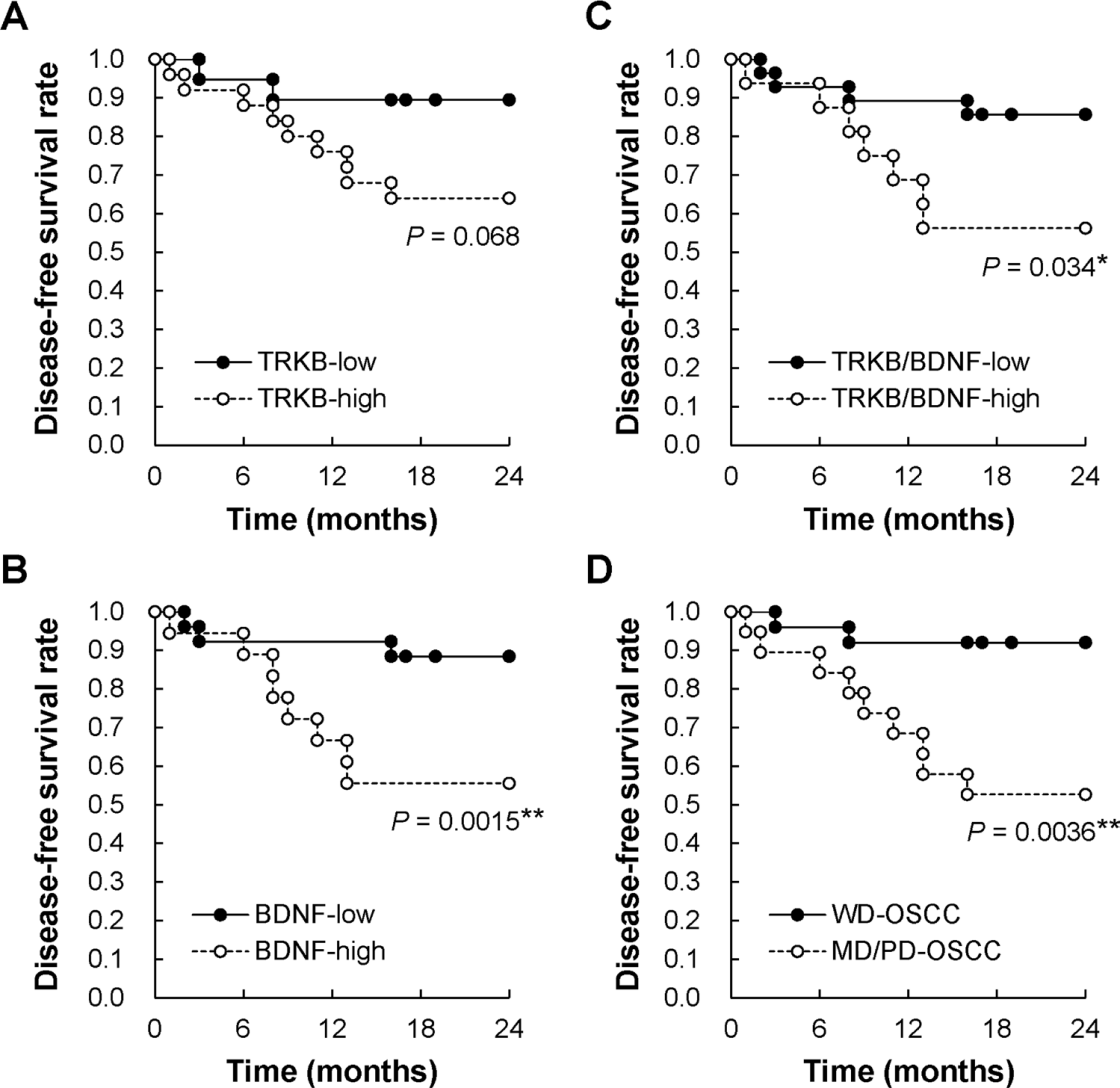
Two-year disease-free survival rate of patients with OSCC Relationships between 2-year disease-free survival and (**A**) TRKB overexpression, (**B**) BDNF overexpression, (**C**) TRKB/BDNF overexpression, and (**D**) tumor differentiation were analyzed using the Kaplan–Meier method. ^*^P < 0.05.

Because almost all patients in this study were classified into stage I and II, which do not involve metastasis, we could not examine the relationship between the expression levels of TRKB, BDNF, or both, and metastasis. The recurrence of metastasis after surgical treatment is known to be present in approximately 20–30% of OSCC at T1 and T2 stages [6, 39]. To investigate this relationship, we assessed the correlations between 2-year disease-free survival and expression levels of TRKB, BDNF, or both, as well as tumor cell differentiation, using the Kaplan–Meier method (Figure 2). As a result, we found that the disease-free survival rate was obviously, but not statistically, lower in patients with TRKB^high^ OSCC compared with those with TRKB^low^ OSCC (*P* = 0.068) (Figure 2A). The survival rates were significantly lower in BDNF^high^, TRKB^high^/BDNF^high^, and MD/PD-OSCC compared to that in controls (*P* = 0.0015, 0.034, and 0.0036, respectively) (Figure 2B–2D). These data show that TRKB^high^ and/or BDNF^high^, as well as moderately/poorly differentiations, represent risk factors for OSCC recurrence and metastasis.

### Significantly higher expressions of TRKB and BDNF in MD/PD-OSCC

**Figure 3:**
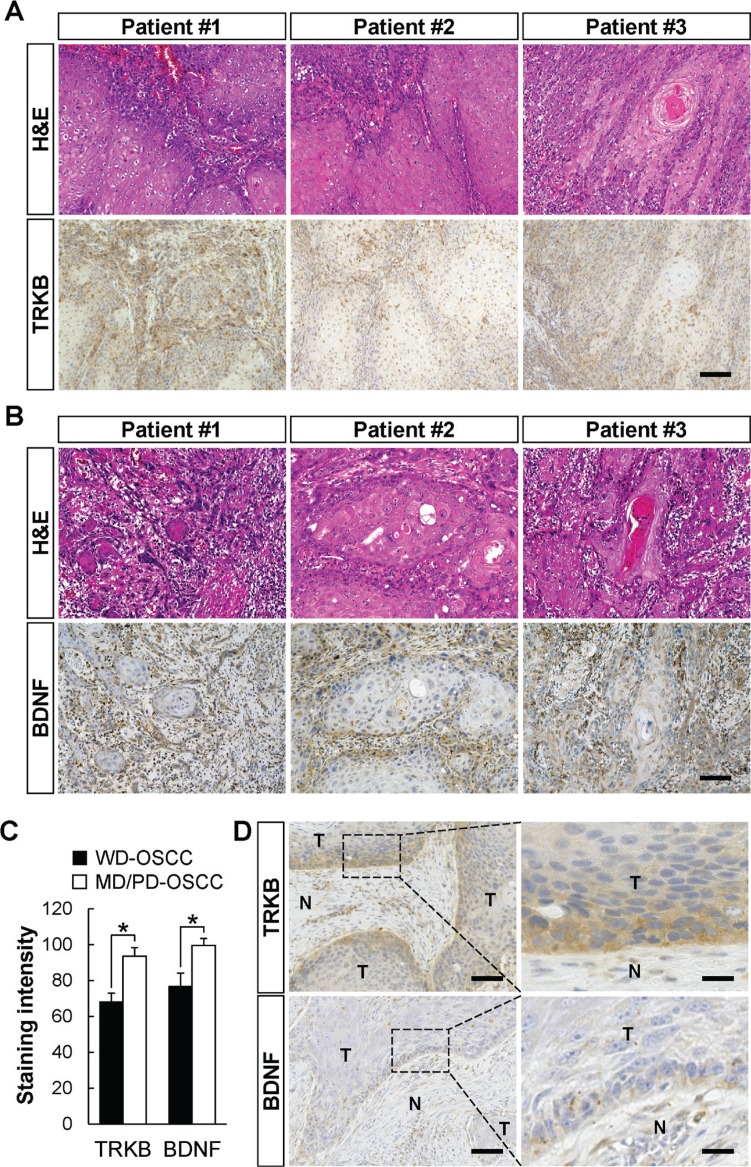
Elevated expressions of TRKB and BDNF in moderately or poorly differentiated human OSCC cells (**A** and **B**) H&E staining and immunohistochemical detection of TRKB (A) and BDNF (B) in well differentiated grade OSCC obtained from the same patients (TRKB, *n* = 6; BDNF, *n* = 4). The representative images from three cases are shown. Bars: 100 μm. (**C**) The staining intensities of TRKB and BDNF in the tumors are shown in histograms with means ± SEM. Open bars and filled bars show the WD-OSCC and MD/PD-OSCC tumors, respectively. ^*^P < 0.05. (**D**) Representative images of immunohistochemical detection of TRKB and BDNF in the invasive front of WD-OSCC tumors. Tumoral (T) and non-tumoral (N) areas are indicated. Bars represent 100 μm (left panels) and 25 μm (right panels).

In Tables 1, 2, and 3, several well differentiated OSCCs showed higher expression of TRKB and/or BDNF. Although OSCC is clinicopathologically classified as either well, moderately, or poorly differentiated, these tumor grades are often mixed together within the classification. We hypothesized that minor PD areas in well differentiated grade OSCC might highly express TRKB and/or BDNF, and exhibit invasive and metastatic phenotypes. To confirm this hypothesis, we examined the expression levels of TRKB, BDNF, or both in WD or MD/PD areas, in specimens obtained from patients with WD-OSCC. As shown in Figure 3, the expression level of the TRKB protein was significantly higher in minor MD/PD areas than in major WD areas in the specimens collected from WD-OSCC patients (Figure 3A and 3C). The same pattern was observed for BDNF (Figure 3B and 3C). Interestingly, the higher expressions of TRKB and/or BDNF were found at the marginal areas of the WD-OSCC tumors (Figure 3D and Supplementary Figure 3). On the other hand, although the expression of other TRK family members, TRKA and TRKC, was tend to be elevated in the marginal area of WD-OSCC tumor and in MD/PD-OSCC tumor, the expression was not significantly different between in WD-OSCC and in MD/PD-OSCC. Their expression levels in some regions of WD-OSCC tumors were as higher as in MD/PD-OSCC tumors (Supplementary Figure 4), although TRKB expression level was clearly and significantly higher in MD/PD-OSCC tumor cells than in WD-OSCC tumor cells. These data indicate that MD/PD-OSCC tumor cells, overexpressing TRKB and/or BDNF, might contribute to metastasis in both MD/PD- and WD-OSCC patients.

### Characterization of human OSCC cell lines

**Figure 4:**
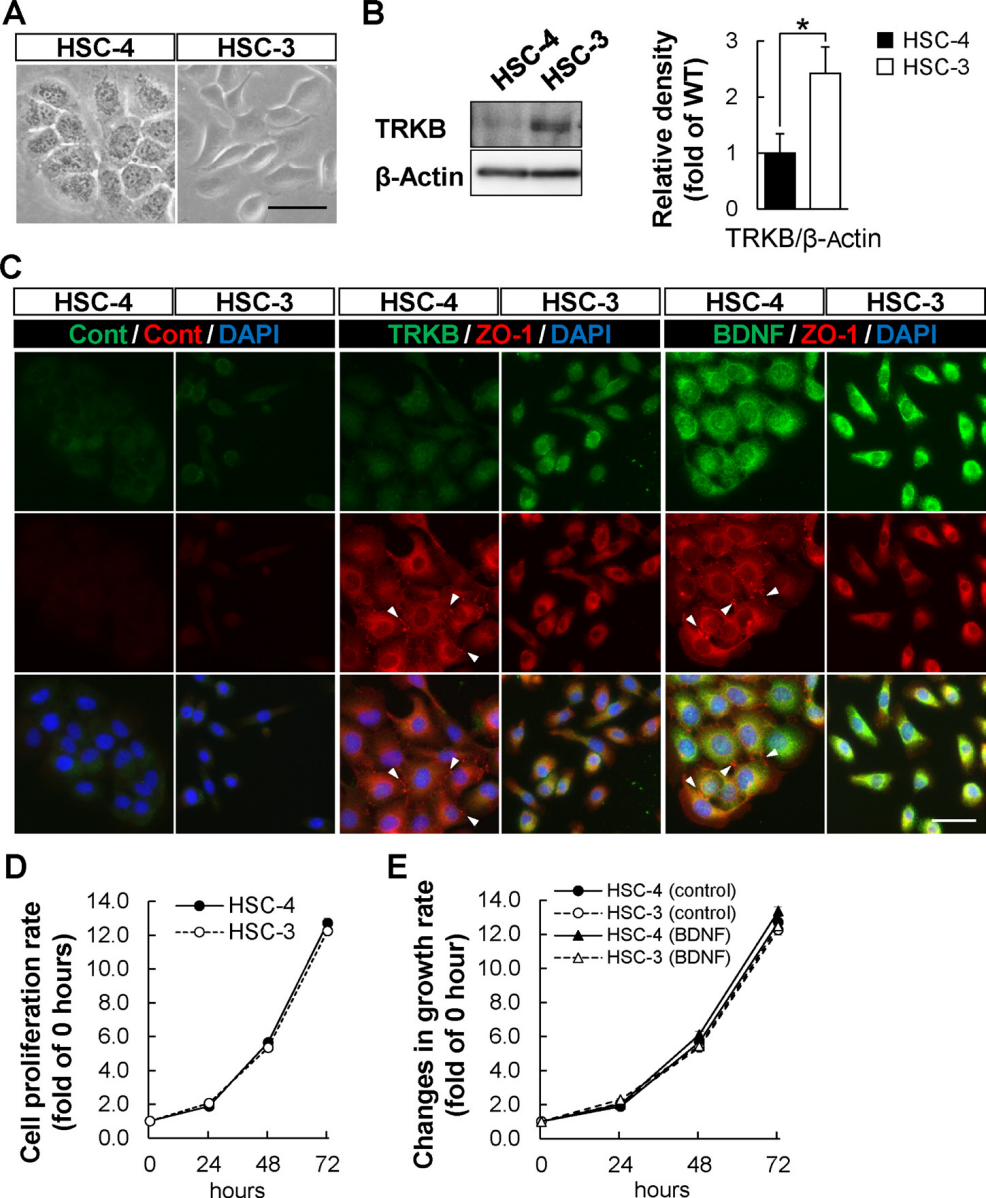
Higher expression of TRKB in HSC-3 poorly differentiated OSCC cell line (**A**) Phase contrast images of HSC-4 (well differentiated) and HSC-3 (poorly differentiated) human OSCC cell lines. Bar: 50 μm. (**B**) TRKB expression levels in HSC-4 and HSC-3 cells were determined by Western blot analysis using anti-TRKB antibody. Histograms show the means ± SEM (*n* = 4 in each cell line) of the densitometry analyses of the blot (HSC-4, filled bar; HSC-3, open bar) as a ratio against control value. β-Actin was used as the loading control. ^*^P < 0.05. (**C**) TRKB and BDNF expressions in HSC-4 and HSC-3 cells were analyzed by immunofluorescence using indicated antibodies. Arrowheads indicate cell-cell junctions visualized by ZO-1-staining. Bar: 50 μm. (**D** and **E**) Cell proliferation assays were performed using HSC-4 and HSC-3 cells cultured for 72 hours in the presence (E) or absence (D) of BDNF (500 ng/mL), following the manufacturer's protocol (see Materials and Methods). Histograms show growth curves of HSC-4 and HSC-3 cells. Data are means ± SEM of triplicated wells from three independent experiments. HSC-4, solid line with filled circle; HSC-3, dashed line with open circle; HSC-4 (BDNF), solid line with filled triangle; HSC-3 (BDNF), dashed line with open triangle.

According to the above analyses of the tumor tissues from the patients with OSCC, the expression levels of TRKB, BDNF, or both were higher in MD/PD-OSCC tumors than in WD-OSCC tumors. To investigate this phenomenon in detail, these correlations were analyzed using two different types of human OSCC cell lines, HSC-4 (WD-OSCC) and HSC-3 (PD-OSCC) [40]. First, these cells were characterized by analyzing cell morphology and the expression of epithelial and mesenchymal markers, using phase and immunofluorescence microscopy (Figure 4A and Supplementary Figure 5A). HSC-4 cells showed a typical epithelial morphology, and expressed E-cadherin and ZO-1 (a scaffolding molecule in adherens junction or tight junction) at cell-cell junctions. HSC-4 cells also expressed low levels of SLUG transcription factor, an epithelial-mesenchymal transition marker. In contrast, HSC-3 cells had a fibroblastic morphology, expressed mesenchymal markers such as SLUG and Vimentin, and showed loss of E-cadherin and ZO-1 at cell-cell junctions. Expression of SNAIL was undetectable in both HSC-4 and HSC-3 cells. Similar data were also obtained through Western blot analysis (Supplementary Figure 5B). The expression of E-cadherin was significantly higher in HSC-4 cells, whereas Vimentin and SLUG were expressed at significantly higher levels in HSC-3 cells. These data showed that HSC-4 and HSC-3 cells displayed epithelial and mesenchymal cell-like phenotypes, respectively.

Next, we investigated whether tumor differentiation was correlated with the expression levels of TRKB and BDNF, using these tumor cell lines (Figure 4B and 4C). Western blot analysis showed that TRKB expression was significantly higher in HSC-3 than in HSC-4 cells. Immunofluorescence analysis showed that the TRKB and BDNF expressions were both higher in HSC-3 than in HSC-4 cells. These data indicate that HSC-3 cells express high levels of TRKB and BDNF, whereas HSC-4 cells express low levels of them. We used HSC-4 as WD-OSCC (TRKB^low^/BDNF^low^), and HSC-3 as PD-OSCC (TRKB^high^/BDNF^high^) in the following experiments.

In addition, to examine the cell proliferation rate and reactivity toward the TRKB-specific ligand, BDNF, in HSC-3 and HSC-4 cells, we performed a cell proliferation assay in Figure 4D and 4E. However, there was no difference between the cells in terms of cell proliferation rate both under normal growth conditions and BDNF-stimulated conditions. Because HSC-3 cells secrete endogenous BDNF, it might be difficult to observe additive effect of BDNF on cell proliferation in HSC-3 cells in this study.

### Correlation between the expression levels of TRKB/BDNF and tumor differentiation in an orthotopic transplantation mouse model of OSCC cell lines

**Figure 5:**
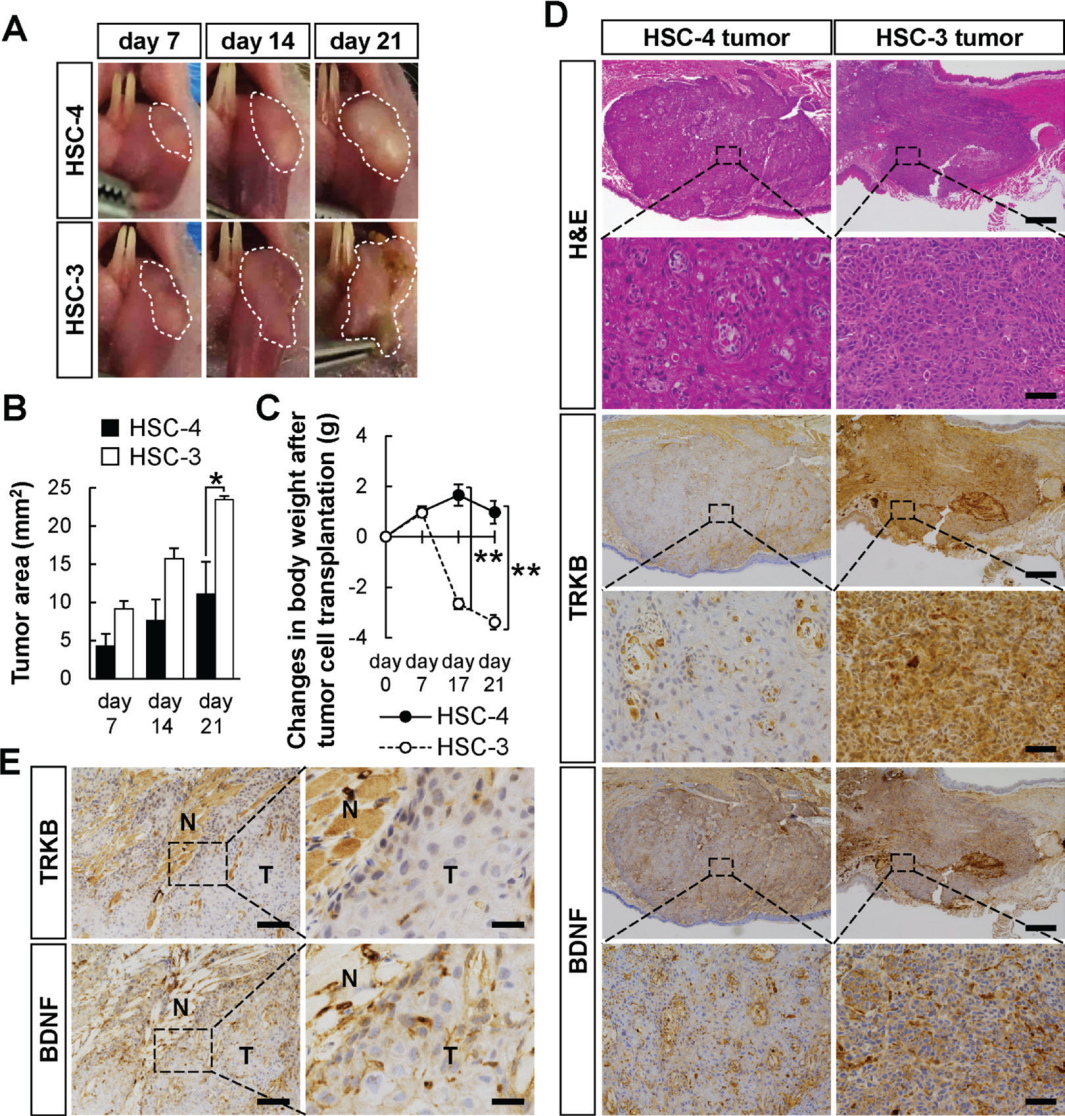
Higher tumor growth of HSC-3 cells orthotopically transplanted in nude mice The difference in tumor growth between HSC-4 and HSC-3 cells was examined by orthotopic transplantation into BALB/cSlc-*nu/nu* mice. (**A** and **B**) The tumor growth area in the tongues was photographed every week (A). Histograms show the means ± SEM (HSC-4, filled bar; HSC-3, open bar; *n* = 3 in each group) of the tumor growth area (mm^2^) (B). ^*^P < 0.05. (**C**) Changes in body weight after the tumor cell transplantation. The body weights were measured at the indicated day points (HSC-4, solid line with filled circle, *n* = 4; HSC-3, dashed line with open circle, *n* = 6). ^**^P < 0.01. (**D**) Representative H&E staining and immunohistochemical detection of TRKB and BDNF in the tumor tissues. Representative images of HSC-4 and HSC-3 tumors are shown. Bars represent 500 μm (low magnification) and 100 μm (high magnification). (**E**) Representative images of immunohistochemical detection of TRKB and BDNF in the invasive front of HSC-4-derived tumors. Tumoral (T) and non-tumoral (N) areas are indicated. Bars represent 100 μm (left panels) and 25 μm (right panels).

To examine the differences in tumor growth *in vivo* between HSC-4 and HSC-3, an orthotopic transplantation of these cells into the tongues of BALB/cSlc-*nu/nu* mice was performed (Figure 5). HSC-4 and HSC-3 cells were submucosally injected into the left side of the tongue of each mouse, and tumor area was analyzed every week after transplantation. HSC-3 cells spread more rapidly in the tongues than HSC-4 cells (Figure 5A and 5B). The body weight of the mice was significantly lower in the HSC-3 cells-transplanted mice than in the HSC-4 cells-transplanted mice (Figure 5C). According to previous reports, a greater prevalence of pain occurs in patients with OSCC than in patients with other cancer types, and the BDNF/TRKB signaling pathway is involved in OSCC-induced pain [41]. The significantly decreased body weight in the HSC-3 cells-transplanted mice might be due to the reduced food and water intake, because of the pain induced by the invasion of HSC-3 cells, in which BDNF and TRKB were highly expressed (Figure 4). Ulcer formation was observed in HSC-3-derived tumors, but not in HSC-4-derived tumors (Figure 5A), which may also be one of the causes.

Next, to examine the tumor differentiation and the TRKB/BDNF expressions in this transplantation model, H&E staining and immunohistochemistry were performed using the tumor tissues 21 days after transplantation (Figure 5D). HSC-4 and HSC-3 cells retained their WD- and PD-morphologies *in vivo*, respectively. HSC-3-derived tumors expressed higher levels of TRKB and BDNF than HSC-4-derived ones. These data indicate that higher expressions of TRKB and BDNF may represent one of the features of PD-OSCC. In Figure 5E, the higher expressions of TRKB and BDNF were also found at the marginal areas of the HSC-4-derived tumors as to WD-OSCC tumors in human specimens (Figure 3D). As shown in Supplementary Figure 6, MMP-9-staining showed that these elevated expressions were seen at the invasive front of tumors in mice.

### Selective reduction of BDNF^high^/TRKB^high^ tumor growth in an orthotopic transplantation mouse model by a TRKB specific inhibitor

**Figure 6:**
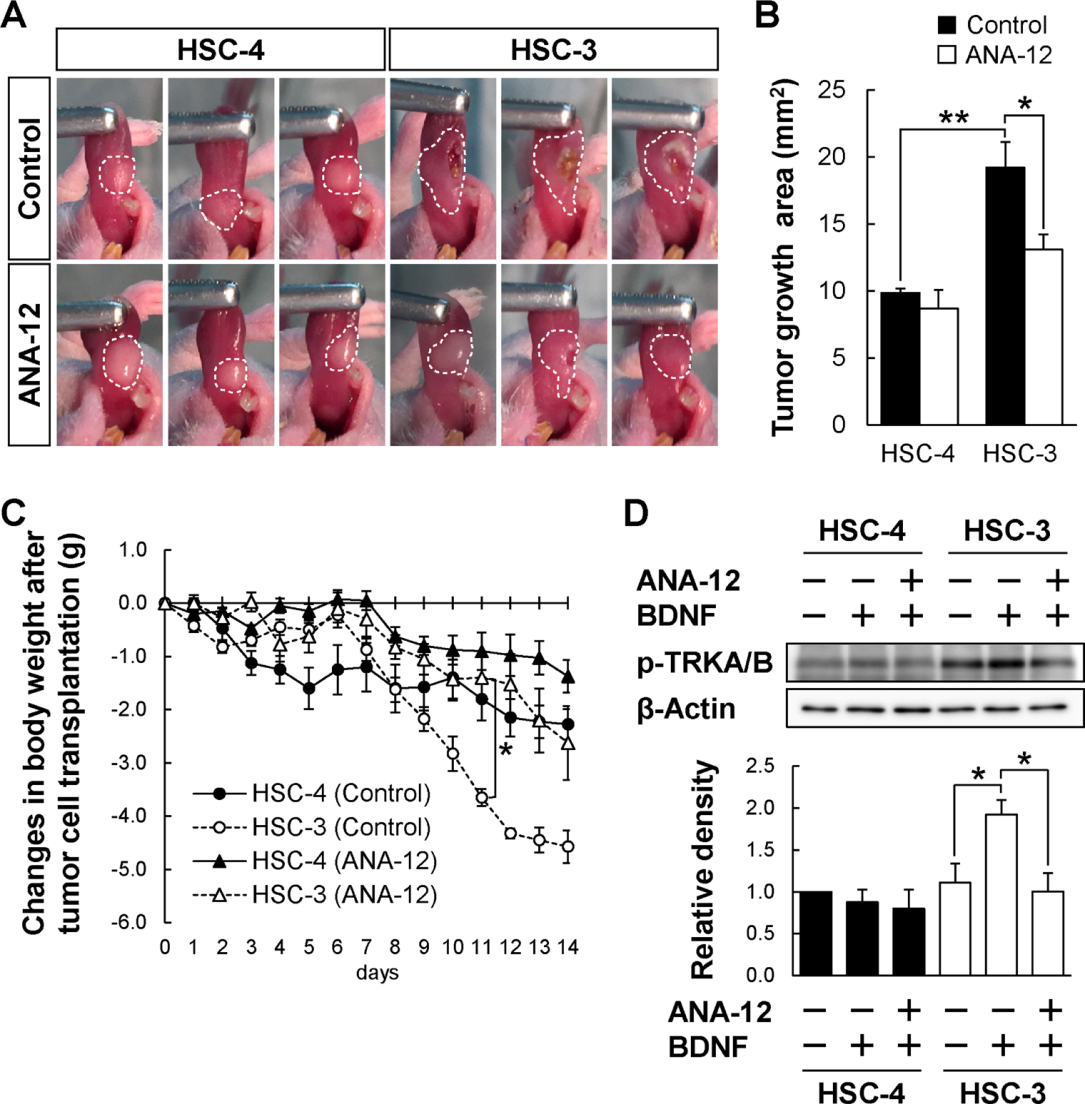
Reduced HSC-3 tumor growth in mice administrated a TRKB-specific inhibitor (**A**–**C**) The effect of ANA-12, a specific TRKB inhibitor, on the tumor growth of HSC-4 and HSC-3 tumor cells *in vivo*. BALB/cSlc-*nu/nu* mice were orthotopically transplanted with these two tumor cell lines and intraperitoneally administrated control (DMSO) or ANA-12 (0.5 mg/kg, every 12 hours) 24 hours after the transplantation. The tumor growth areas in the tongues were photographed at day 14 after the transplantation (A). Histograms show the means ± SEM (control, filled bar; ANA-12, open bar; *n* = 4 in each group) of the tumor growth area (mm^2^) (B). ^**^P < 0.01 and ^*^P < 0.05. (C) Changes in body weight after the tumor cell transplantation. The body weights were measured every day after the transplantation (control, solid line; ANA-12, dashed line; HSC-4, filled circle or triangle; HSC-3, open circle or triangle; *n* = 4 in each group). ^**^P < 0.01. (**D**) The effect of ANA-12 on the BDNF-induced phosphorylation of TRKB was examined by Western blot analysis using anti-phospho-TRKA/B antibody. Cultured HSC-4 and HSC-3 cells were pre-treated with ANA-12 (10 μM) for 1 hour, and subsequently cultured for 20 minutes in the presence or absence of BDNF (100 ng/mL). β-Actin was used as the loading control. Histograms show the means ± SEM (three independent experiments) of the densitometry analyses of the blot (HSC-4, solid bar; HSC-3, filled bar) as a ratio against control value. Note: BDNF-induced TRKA/B phosphorylation and suppression of the phosphorylation were observed only in HSC-3 cells but not in HSC-4 cells. ^*^P < 0.05.

To investigate whether TRKB-selective inhibition could selectively suppress BDNF^high^/TRKB^high^ PD-OSCC tumor growth *in vivo*, orthotopic transplantation of HSC-4 or HSC-3 into the tongues of BALB/cSlc-*nu/nu* mice was performed in the presence or absence of a TRKB specific inhibitor, ANA-12 (Figure 6). Twenty-four hours after the tumor transplantation, some mice were peritoneally injected with ANA-12 every 12 hours and weighed daily. The tumor growth was analyzed at 14 days after the transplantation. As shown in Figure 6A and 6B, only BDNF^high^/TRKB^high^ HSC-3-derived tumor growth was significantly reduced by ANA-12 treatment. The reduction in body weight of HSC-3-transplanted mice was significantly blocked by ANA-12 treatment (Figure 6C). Admittedly, ulcer formation in the HSC-3-derived tumor was suppressed by ANA-12 treatment (Figure 6A). The HSC-3-transplanted mice without ANA-12 treatment gathered together and hardly moved in the cage, while the mice treated with ANA-12 actively moved around the cage, similar to HSC-4-transplanted mice with or without ANA-12 treatment (Supplementary Figure 7).

We therefore investigated whether the BDNF/TRKB signaling pathway could work differently in either HSC-3 or HSC-4, and whether ANA-12 could selectively suppress BDNF-dependent TRKB activation, using Western blot analysis on *in vitro*-cultured HSC-4 and HSC-3 cells. BDNF induces phosphorylation of TRKB (Tyr 532), which requires TRKB activation, subsequent growth, and migration signal transduction [29]. Tumor tissue in the tongue is not suitable for Western blotting, because TRKB and BDNF are also expressed in tongue muscle and inflammatory cells. As shown in Figure 6D, BDNF-dependent increase of TRKB phosphorylation was only observed in HSC-3 cells, but not in HSC-4 cells, and the increase was hampered by the treatment with ANA-12, a TRKB-specific inhibitor. These data suggest that the blockage of TRKB activation by a TRKB-specific inhibitor could selectively suppress BDNF^high^/TRKB^high^ PD-OSCC tumor growth.

### Selective reduction of BDNF^high^/TRKB^high^ PD-OSCC cell migration and tumor growth by a TRKB-specific inhibitor

**Figure 7:**
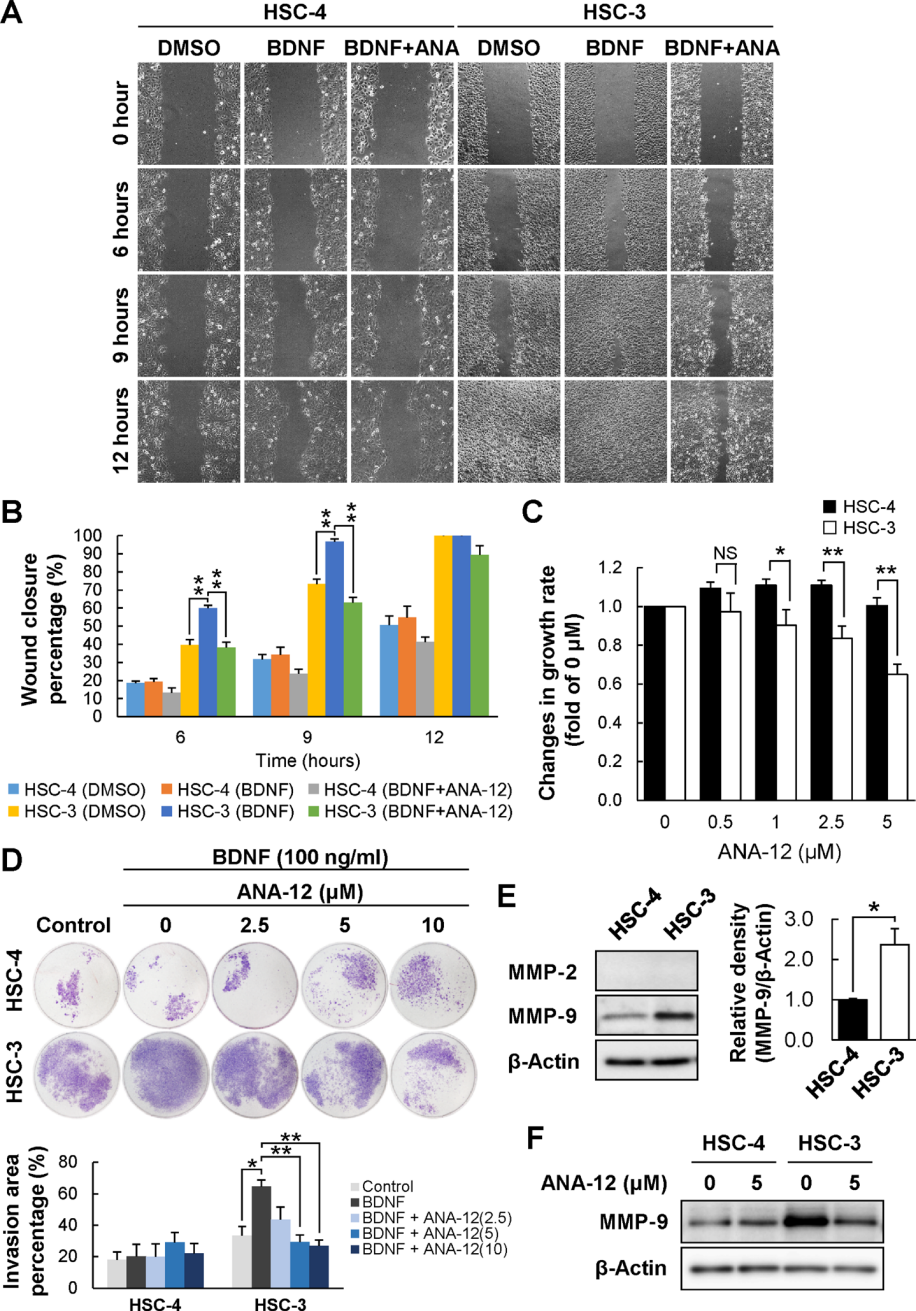
Reduced cell migration and proliferation of HSC-3 following treatment with a TRKB-specific inhibitor The effects of ANA-12 on cell migration and proliferation were examined by wound healing assay (**A** and **B**) and WST-8 cell proliferation assay (**C**), respectively. (A and B) Wound healing assays were performed using HSC-4 and HSC-3 treated with BDNF (100 ng/mL) in the presence or absence of ANA-12 (10 μM) for 12 hours. The migrating cells were photographed every 3 hours (A) and cell migration was estimated as wound closure percentage (B). Histograms show the means ± SEM of the percentages of wound closure at 6, 9, and 12 hours. ^**^P < 0.01 and ^*^P < 0.05. NS, not significant. (C) Cell proliferation assays were performed using HSC-4 and HSC-3 cells cultured for 72 hours in the presence or absence of ANA-12 (0, 0.5, 1, 2.5, and 5 μM), following the manufacturer's protocol (see Materials and Methods). Histograms show changes in growth rate relative to control (0 μM) as the means ± SEM of triplicated wells from at least three independent experiments (HSC-4, *n* = 6; HSC-3, *n* = 3). Filled bars and open bars show HSC-4 and HSC-3 cells, respectively. ^**^P < 0.01 and ^*^P < 0.05. NS, not significant. (**D**) Transwell Matrigel invasion assays were performed using HSC-4 and HSC-3 cells cultured for 24 hours in the presence or absence of ANA-12 (0, 2.5, 5, 10 μM). Histograms show the percentage of invasion area to filter as the means ± SEM of three independent experiments. ^**^P < 0.01 and ^*^P < 0.05. (**E**) Expression levels of MMP-2 and MMP-9 in these cells were determined by Western blot analysis. Histograms show the means ± SEM (*n* = 3 in each cell line) of the densitometry analyses of the blot (HSC-4, filled bar; HSC-3, open bar) as a ratio against control value. β-Actin was used as the loading control. ^*^P < 0.05. (**F**) The cells were cultured for 24 hours in the presence or absence of ANA-12 (5 μM) and the effect of ANA-12 on MMP-9 expression in these cells were examined by Western blot analysis.

The BDNF/TRKB signaling pathway mediates tumor cell growth, migration, and invasion. First, we examined the effect of ANA-12 on cell migration of HSC-3 and HSC-4 by a wound healing assay (Figure 7A and 7B). The rate of wound healing in HSC-3 cells was significantly higher than in HSC-4 cells. Despite no effect of BDNF and ANA-12 on HSC-4 cell migration, HSC-3 cell migration was significantly upregulated by BDNF stimulation, and the upregulation was completely hampered by ANA-12 treatment. Next, we examined the effect of ANA-12 on the growth of HSC-3 and HSC-4 cells, and the results obtained were similar to those observed in the cell proliferation assay (Figure 7C). The growth of HSC-4 was not affected by ANA-12. In contrast, a significant dose-dependent reduction of growth was observed in HSC-3 cells in the presence of ANA-12. Moreover, we examined the effect of ANA-12 on the tumor cell invasion of these cells using Transwell Matrigel invasion assay (Figure 7D). The invasion of HSC-3 cells was upregulated by BDNF stimulation and the upregulation was dose-dependently suppressed by ANA-12 treatment, whereas that of HSC-4 cells was not affected by BDNF or ANA-12 treatment. Controlled remodeling of extracelluar matrix is essential for tumor cell invasion and regulated by matrix metalloproteinases (MMPs). In Figure 7E and 7F, Western blot analysis showed that HSC-3 cells expressed higher levels of MMP-9 compared to HSC-4 cells and the expression of MMP-9 in HSC-3 cells was downregulated in the presence of ANA-12. Expression of MMP-2 in the both cells was under detection level in our study. In Supplementary Figure 8A and 8C, treatment of HSC-3 cells with ANA-12 caused a change in cell morphology from fibroblast-like, spindle-shaped to epithelial-like form, downregulated SLUG, and upregulated E-cadherin (Supplementary Figures 8B, 8D, and 9), suggesting that ANA-12 treatment induced mesenchymal-epithelial transition, MET in HSC-3 cells. Taken together, these data suggest that selective blockage of the BDNF/TRKB signaling pathway by a TRKB-specific inhibitor could selectively suppress cell proliferation, migration, and invasion of BDNF^high^/TRKB^high^ PD-OSCC tumor cells.

## DISCUSSION

Although *NTRK2* (originally identified as *OncD*), which encodes TRKB, is an old oncogene identified 35 years ago [17–19], no drug targeting this molecule has developed yet for cancer therapy. The molecular functions of TRKB have been investigated using tumor cell lines and tumor transplantation mouse model systems; furthermore, the relationship between the expression and clinical significance in patients with solid tumors has been established. Recently, several TRK-targeting compounds have reached the clinical development stage [7, 21, 22, 42]. However, the type of OSCC in which TRKB signaling aberrantly works, and the relationship between TRKB expression level and clinical features in patients with OSCC, are not yet fully understood. In this study, we describe a new relationship between TRKB/BDNF expression and tumor differentiation in patients with OSCC, and propose TRKB as a potential therapeutic target for OSCC, especially for PD-OSCC. First, the expression levels of TRKB and/or its specific ligand, BDNF, were significantly higher in MD/PD-OSCC tumor cells than in WD-OSCC tumor cells. TRKB^high^ and/or BDNF^high^ OSCC significantly increased tissue invasion and lymph node metastasis. An orthotopic transplantation mouse model experiment, with two types of human OSCC cell lines, showed that the administration of a TRKB-specific inhibitor significantly reduced tumor growth or invasion in HSC-3 (PD-OSCC, TRKB^high^/BDNF^high^)-derived tumors, but not in HSC-4 (WD-OSCC, TRKB^low^/BDNF^low^)-derived tumors. Our data support the idea that BDNF/TRKB signaling may regulate tumor progression in OSCC, especially PD-OSCC, and that high expression of these molecules may be an attractive prognostic marker for tumor aggressiveness, as well as a potential target for OSCC therapies.

OSCC is one of the most common and lethal head and neck malignancies worldwide, and many patients with OSCC have a poor response to therapy, as well as high recurrence rates due to metastasis. The recurrence rate after surgical treatment is approximately 20–30% at T1/T2 stages and 50–60% at T3/T4 stages [6, 39]. Cisplatin and cetuximab are often used for OSCC treatment, in combination with radiation, and significantly prolong progression-free survival in patients with OSCC. However, these drugs show no significant improvement in terms of complete recovery. One of the reasons for that is tumor heterogeneity, which contributes to tumor drug resistance, metastasis, and recurrence. Therefore, a deeper understanding of OSCC's molecular characteristics is needed to establish a more accurate classification process, and provide more efficient drug therapies to OSCC patients.

In this study, we revealed that the BDNF/TRKB signaling pathway, unlike other pathways related to the TRK family (TRKA and TRKC), contributes to OSCC tumor growth and invasion and therefore, identified these molecules as markers of MD/PD-OSCC tumor cells, which tend to infiltrate and metastasize into surrounding tissues. Previously, in patients with neuroblastoma, a study showed that high expressions of TRKA or TRKC in the tumor had no influence on the quality of the prognosis, whereas poor prognosis was observed when high expressions of TRKB and/or BDNF occurred [29], which is consistent with our results.

Regarding the mechanisms driving TRKB and BDNF upregulation in tumor cells, hypoxia is thought to be one of the key factors in the tumor environment. Hypoxia induces increases in *NTRK2* (TRKB) gene expression that are mediated by HIF-1α binding to a hypoxia response element in the *NTRK2* promoter region. Moreover, hypoxia-associated increases in neuroblastoma cell invasiveness can be reversed by blockage of the TRK-kinase activity [43]. These findings suggest that hypoxia increases TRKB and BDNF expressions, and consequently functions in the tumor microenvironment as the autocrine activator of the BDNF/TRKB signal pathway, enabling tumor cell proliferation, invasion, metastasis, and survival after multiple rounds of chemotherapy. The BDNF/TRKB signaling pathway contributes to the acquisition of resistance to a variety of chemotherapy agents through the activation of the PI3K/AKT survival pathway [44, 45]. From our study, the analysis of the relationship between 2-year disease-free survival and high expression of TRKB/BDNF showed significantly higher rates of tumor recurrence in patients with TRKB^high^ and/or BDNF^high^ OSCC than in those suffering from TRKB^low^/BDNF^low^ OSCC (Figure 2).

We also observed high expressions of both TRKB and BDNF in tumor-associated vessels, even though these vessels appeared to be normal and were located far from the tumor cells in OSCC patients (Supplementary Figure 2). We also demonstrated high expressions of these proteins in neo-vessels of both HSC-4 and HSC-3 cell-derived tumors in the mouse transplantation model systems (data not shown), raising the possibility that OSCC tumor cells secreting BDNF are able to induce tumor angiogenesis. Previously, the existence of a direct control of TRKB and BDNF on angiogenesis was reported. TRKB-deficient mice showed a marked reduction in blood vessel density and an increased number of TUNEL-positive apoptotic endothelial cells. TRKB and BDNF are upregulated in vascular vessels at certain developmental stages. As with TRKB, BDNF is also significantly induced in ischemic conditions compared to that in non-ischemic ones, and this high expression promotes blood flow recovery and capillary density through TRKB [46, 47]. These findings indicate that the BDNF/TRKB signaling pathway functions not only in tumor cells, but also in tumor-associated vessels, suggesting the involvement of this pathway in multiple processes of tumor progression, including tumor cell proliferation, invasion, metastasis, and angiogenesis.

It was reported that TRKB and BDNF are both highly expressed in more than 50% of human head and neck squamous cell carcinoma (NHSCC) tumors [32], and that the positive expression rates of TRKA, TRKB, and TRKC in human OSCC reach 90.2% (92/102 cases), 31.4% (32/102 cases), and 47.0% (48/102 cases), respectively [37]. Our data, which showed the frequent expression of TRK family members in OSCC tumors (TRKA, 13/15 cases, 86.7%; TRKB, 36/44 cases, 79.5%; TRKC, 14/15 cases, 93.3%), are consistent with these reports, indicating the potential usefulness of TRK inhibitors in most OSCC tumors.

In our *in vitro* and/or *in vivo* assays, tumor cell growth and migration were blocked by a TRKB-specific inhibitor in HSC-3 TRKB^high^/BDNF^high^ PD-OSCC tumor cells. Although the blockage was not complete, the TRKB-specific inhibitor could effectively hamper growth and migration of high metastatic TRKB^high^/BDNF^high^ PD-OSCC tumors. Because metastasis was not observed in HSC-3-transplanted mice models, we could not investigate the effect of a TRKB-specific inhibitor on tumor metastasis in this study. Further experiments should be performed to clarify the effect of the inhibitor on tumor metastasis *in vivo*. Given that TRKB is highly expressed in MD/PD-OSCC, TRKB selective blockage could efficiently suppress the growth of MD/PD-OSCC tumor, allowing for a better prognosis in patients with OSCC. Combined therapy of TRKB inhibitor with standard drugs, such as cetuximab and cisplatin, could suppress OSCC tumors more effectively, resulting in complete remission.

In summary, we report here a new correlation between TRKB/BDNF overexpression and OSCC tumor differentiation using human tissue specimens, and propose TRKB as an attractive therapeutic target for OSCC. Our data suggest that the BDNF/TRKB signaling pathway may mediate OSCC tumor progression, especially multiple behaviors of aggressive MD/PD-OSCC tumor cells. Applying a treatment with a TRKB specific inhibitor, we demonstrate the selective effects of TRKB blockage on TRKB^high^/BDNF^high^ MD/PD-OSCC tumor cell proliferation and migration in *in vitro* or *in vivo* assay systems. Our findings provide insight into a possible therapeutic strategy to prevent invasive and metastatic tumors toward developing future therapies for OSCC.

## MATERIALS AND METHODS

### Patients and tissue specimens

OSCC tumor tissues and adjacent normal tissues were obtained from a total of 44 patients diagnosed with tongue squamous cell carcinoma, who had not received any previous treatment, and had undergone surgery in Osaka Medical College Hospital under an Institutional Review Board-approved protocol. Informed consent was obtained from all patients. The patients comprised 23 men and 21 women aged between 30 and 92 years (mean age of 66.4 years) who had not received any neoadjuvant therapy. Medical records and prognostic follow-up data were obtained from these patients by retrospective chart review.

The tissue specimens from the patients were fixed in 10% buffered formalin, dehydrated in a graded ethanol series, and embedded in paraffin. Serial sections (3-μm-thick) were then prepared, and subsequently stained using hematoxylin and eosin (H&E). All histopathological data were reviewed from the corresponding H&E-stained images. The OSCC tumors were classified according to the pathological Tumor-Node-Metastasis (TNM) classification of the UICC staging system. Tumor differentiation was classified according to the World Health Organization (WHO) histological classification system. Clinicopathological information on the patients, including anatomic subsite of the oral cavity, tumor size, stage, histopathological findings of tumor differentiation, lymphatic and vascular invasions, and lymph node metastasis was obtained from the medical records of the patients, and summarized in Tables 1–4. Lymphatic and vascular invasions were defined as the presence of aggregates of tumor cells within endothelial lined spaces visualized through podoplanin (clone D2-40, Dako, CA, USA) and CD34 (clone QBEnd 10, Dako) staining, respectively.

### Immunohistochemistry

Immunohistochemistry (IHC) on human OSCC specimens and mouse tumor tissues was performed using a BOND-MAX autoimmunostainer (Leica Microsystems, Wetzlar, Germany). Deparaffinized and rehydrated sections, which contained the deepest site for each tumor, were subjected to endogenous peroxidase blocking. After heating in an antigen unmasking solution, slides were incubated with the following antibodies: TRKB (1:100; sc-8316, Santa Cruz Biotechnology, CA, USA) and BDNF (1:100; LS-B6557, LifeSpan BioSciences, WA, USA). Color development was carried out using diaminobenzidine tetrahydrochloride, and slides were counterstained with hematoxylin. All samples were stained under the same conditions. For negative controls, primary antibodies were omitted. Human brain histological specimens were used as TRKB and BDNF positive controls. For image analysis, these immunostained sections were scanned using a microscope (BZ-X710, Keyence, Osaka, Japan).

### Evaluation of immunohistochemistry

Immunohistochemical detection of TRKB and BDNF in all patients with OSCC was performed using more than three independent high-power microscopic fields (×400) for each OSCC tissue specimen. The staining intensity was ranked from 0 to 3 degrees of staining (0 = none, 1 = weak, 2 = moderate, 3 = strong) and was divided into 2 groups, low (grades 0 and 1) and high (grades 2 and 3). For quantitative assessment of TRKB and BDNF immunohistochemistry in each patient with WD-OSCC, the staining intensity of each specimen was evaluated by staining intensity score. Staining intensity score was calculated using the following formula: staining intensity score = 255 − mean of grey value. The mean of grey value was measured on a scale from 0 (maximum staining) to 255 (no staining) by Image J software, using each stained image.

### Cell culture

HSC-4 and HSC-3 human tongue squamous cell carcinoma cells [40] were purchased from Japanese Collection of Research Bioresources (JCRB) Cell Bank (Osaka, Japan) (JCRB0624 and JCRB0623, respectively), and maintained in Dulbecco's modified Eagle medium, DMEM (Thermo Fisher Scientific, MA, USA) supplemented with 10% fetal bovine serum (FBS) (Invitrogen Japan, Tokyo, Japan) and penicillin/streptomycin (100 IU/50 μg/mL) (Invitrogen Japan) at 37° C in a humidified atmosphere containing 5% CO_2_.

### Western blotting

HSC-4 and HSC-3 cells were cultured with human recombinant BDNF (100 ng/mL) (Peprotech, NJ, USA) for 20 minutes in the presence or absence of ANA-12, a TRKB inhibitor (10 μM), and then lysed in SDS-PAGE sample buffer. These lysates were aliquoted and stored at −80° C until used. Protein concentrations were determined using Coomassie brilliant blue-staining protein spot for each specimen, with a standard concentration of protein. Equivalent amounts (2, 5, or 15 μg) of protein were loaded for each condition and resolved by SDS-PAGE, electrophoretically transferred to PVDF membrane (Merck Millipore, Darmstadt, Germany), blocked with 5% BSA in Tris-buffered saline (TBS), and probed overnight at 4° C with antibodies against TRKB (1:1,000; Santa Cruz Biotechnology), phospho-TRK (1:1,000; mouse TRKA Tyr 490/TRKB Tyr 516; human TRKA Tyr 496/TRKB Tyr 532) (1:1,000; #4619, Cell Signaling Technology Japan), MMP-2 (1:1,000; bs-0412R, Bioss), MMP-9 (1:1,000; 10375-2-AP, Proteintech), and β-Actin (1:20,000; clone AC-15, Sigma-Aldrich Japan). Blots were then washed with TBS containing 0.05% Tween 20 (TBST), incubated for 1 hour with horse radish peroxidase (HRP)-conjugated anti-rabbit IgG or anti-mouse IgG antibodies (1:5,000; Jackson Laboratory) in TBST supplemented with 5% BSA at room temperature, and subsequently washed with TBST. Blots were then developed using a Luminata Western HRP substrate (Merck Millipore). Signals were detected and documented with the densitometry system LAS3000 (Fujifilm, Tokyo, Japan).

### Immunofluorescence staining

HSC-4 and HSC-3 cells were cultured for 48 hours on 0.1% gelatin-coated glass-coverslips, fixed in 4% paraformaldehyde (PFA) in phosphate-buffered saline (PBS) for 30 minutes, permeabilized for 10 minutes with 0.1% Triton X-100 in PBS, and blocked with 3% bovine serum albumin (BSA) (Nacalai Tesque, Kyoto, Japan) in PBS for 1 hour at room temperature. These cells were then stained with primary antibodies against SLUG (1:200; #9585, Cell Signaling Technology Japan, Tokyo, Japan), E-cadherin (1:200; #3195, Cell Signaling Technology Japan), Vimentin (1:200; #5741, Cell Signaling Technology Japan), ZO-1 (clone T8-754, kindly provided by Dr. Masahiko Ito, Dokkyo Medical University, Japan and Dr. Mikio Furuse, National Institute for Physiological Sciences, Japan), TRKB (1:200; Santa Cruz Biotechnology), and BDNF (1:200; LifeSpan BioSciences) for 3 hours at room temperature. The following secondary antibodies were subsequently used: Alexa Fluor488-conjugated donkey anti-rabbit IgG (1:500; Invitrogen Japan) or Cy3-conjugated donkey anti-mouse IgG (1:500; Jackson Laboratory, ME, USA) for 1 hour at room temperature. Control rabbit and mouse IgG were purchased from Chemicom International, Inc. (CA, USA) and Upstate Biotechnology (NY, USA), respectively. DAPI (0.1 μg/ml; 4′,6-diamidino-2-phenylindole; Invitrogen Japan) counterstaining was also performed. Stained specimens were examined using a fluorescence microscope, BZ-X710.

### Cell proliferation assay

HSC-4 and HSC-3 cells were trypsinized and seeded on 96-well culture plates at a density of 3,500 and 3,000 cells per well, respectively. The cells were cultured with dimethyl sulfoxide (DMSO) (Nacalai Tesque) or BDNF (500 ng/ml) in the presence or absence of ANA-12 (0, 0.5, 1, 2.5, and 5 μM) for 72 hours. The proliferation assay was performed using a disulfonated tetrazolium salt, WST-8 (Dojindo, Kumamoto, Japan), following the manufacturer's instructions. Briefly, at the indicated time points, WST-8 was added to each well and the cells were incubated for 3 hours at 37° C. The absorbance values for each well were measured at 450 nm on the iMark Microplate Reader (Bio-Rad Laboratories, CA, USA).

### Orthotopic transplantation mouse model of tongue squamous cell carcinoma

BALB/cSlc-*nu/nu* female mice were purchased from Japan SLC (Shizuoka, Japan) and housed in a laminar air-flow cabinet, in a barrier facility, under specific pathogen-free conditions. For transplantation, HSC-4 or HSC-3 cells were dissociated with Trypsin (0.05%)/EDTA (0.02%), and single-cell suspensions were prepared in sterile PBS. The viability of the cells was checked before injection (>95%). Under 2,2,2-tribromoethanol (Sigma-Aldrich Japan, Tokyo, Japan) anesthesia, 8-week-old BALB/cSlc-*nu/nu* mice were injected submucosally with 1.75 × 10^5^ HSC-4 or HSC-3 cells (30 μL) into the left side of the tongue using a Hamilton syringe with a 26-gauge needle. A TRKB specific inhibitor, ANA-12 (Abcam, Cambridge, UK), was administered at 0.5 mg/kg into each mouse intraperitoneally 24 hours after the transplantation. The injections were performed every 12 hours for 20 days. The body weight and tumor size were measured every day and every week, respectively. The area occupied by the tumor was photographed and measured using Adobe PhotoShop software. On day 21 after tumor transplantation, these mice were sacrificed, and the tongues isolated, fixed with 4% PFA in PBS overnight at 4° C, embedded in paraffin, and sectioned for histological analysis. All studies on animal models were approved by the Ethical Committee of the Osaka Medical College and performed according to its guidelines.

### Wound healing assay

HSC-4 and HSC-3 cells were seeded on 24-well plates and after reaching subconfluence, the cells were treated with DMSO or ANA-12 (10 μM, 1 hour). Subsequently, the cells were wounded with a sterile 200 μL pipet tip and incubated with BDNF (100 μg/mL) in the presence or absence of ANA-12. Phase contrast images of migrating cells were captured with a 10× objective on a Nikon Eclipse Ti-E microscope with Cell Motion Imaging System (SI8000, Sony Corporation, Tokyo, Japan). Images were processed with Adobe PhotoShop software. Wound closure percentage = [(1–wound area at a certain time point)/starting wound area] × 100.

### Transwell matrigel invasion assay

The invasion assay was designed using Matrigel (#356230, Corning Costar, Cambridge, MA, USA)-coated Transwell plates (#3422, Corning Costar) which were 6.5 mm in diameter with 8 μm pore filters. Briefly, the upper chambers were loaded with HSC-4 or HSC-3 cells (5 × 10^4^ cells/well) in 100 μl of DMEM containing 0.5% FBS, and/or ANA-12 (0, 2.5, 5, 10 μM), and the lower chambers with 600 μl of DMEM containing 0.5% FBS, 100 ng/ml of BDNF, and/or ANA-12. After 24 hours of incubation, cells which had failed to invade were removed from the upper chambers. Those that were attached to the outside of the filter were fixed and stained with 0.2% crystal violet and photographed at a stereoscopic microscopy (SMZ745T, Nikon, Tokyo, Japan) with a digital camera (DS-Vi1, Nikon). The staining area of each filter was measured by Photoshop software and the cell invasion was calculated as percentage of cell coverage to the filter (invasion area percentage). The invasion assays were repeated at three times.

### Statistical analysis

The correlations between the expressions of TRKB or BDNF and clinicopathological variables were evaluated using Pearson's χ^2^ test or Fisher's exact test. The comparison between the two groups was performed using unpaired two-tailed Student's *t*-test. Results are presented as mean ± standard error of the mean (SEM). *P*-values are indicated as follows; ^***^P < 0.001, ^**^P < 0.01, and ^*^P < 0.05.

## SUPPLEMENTARY MATERIALS FIGURES AND TABLES



## Abbreviations

BDNF: brain-derived neurotrophic factor
BSA: bovine serum albumin
DAPI: 4’,6-diamidino-2-phenylindole
DMEM: Dulbecco's modified Eagle medium
DMSO: dimethyl sulfoxide
EGFR: epidermal growth factor receptor
FBS: fetal bovine serum
IHC: Immunohistochemistry
H&E: hematoxylin and eosin
HNSCC: head and neck squamous cell carcinoma
HRP: horse radish peroxidase
MMP: matrix metalloproteinase
OSCC: oral squamous cell carcinoma
MD-OSCC: moderately differentiated OSCC
PD-OSCC: poorly differentiated OSCC
PFA: paraformaldehyde
TBS: Tris-buffered saline
TNM: Tumor-Node-Metastasis
TRK: tropomyosin receptor kinase
WD-OSCC: well differentiated OSCC
WHO: World Health Organization
