# Supplementary material for: TRKB tyrosine kinase receptor is a potential therapeutic target for poorly differentiated oral squamous cell carcinoma
Source: Oncotarget. 2018 May 18;9(38):25225–43. doi: 10.18632/oncotarget.25396 (PMC5982746; doi:10.18632/oncotarget.25396)
Supplement: Supplementary file 1 [file oncotarget-09-25225-s001.pdf]

TRKB tyrosine kinase receptor is a potential therapeutic target for poorly differentiated oral squamous cell carcinoma

SUPPLEMENTARY MATERIALS

Human tissue specimens & Immunohistochemistry

Tissue specimens from the patients with oral squamous cell carcinoma (OSCC) were fixed in 10% buffered formalin, dehydrated in a graded ethanol series, and embedded in paraffin. Serial sections (3-μm-thick) were then prepared, and these were analyzed using hematoxylin and eosin (H&E) staining and immunohistochemistry. Immunohistochemistry was performed using a BOND-MAX autoimmunostainer (Leica Microsystems, Wetzlar, Germany). Deparaffinized and rehydrated sections which contained the deepest site for each tumor were subjected to endogenous peroxidase blocking. After heating in antigen unmasking solution, slides were incubated with the following antibodies: TRKB (1:100; sc-8316, Santa Cruz Biotechnology, CA, USA), BDNF (1:100; LS-B6557, LifeSpan BioSciences, WA, USA), TRKA (1:200; sc-118, Santa Cruz Biotechnology), TRKC (1:400; ab75174, Abcam, Cambridge, UK), and MMP-9 (1:100; 10375-2-AP, Proteintech). Color development was carried out using diaminobenzidine tetrahydrochloride, and slides were counterstained with hematoxylin. All samples were stained under the same staining conditions. For image analysis, these immunostained sections were scanned using a microscope (BZ-X710, Keyence, Osaka, Japan).

Cell culture and phase-contrast images

HSC-4 and HSC-3 human OSCC cells were purchased from Japanese Collection of Research Bioresources (JCRB) Cell Bank (Osaka, Japan) (JCRB0624 and JCRB0623, respectively) and maintained in Dulbecco's modified Eagle medium, DMEM (Thermo Fisher Scientific, MA, USA) supplemented with 10% fetal bovine serum (Invitrogen Japan, Tokyo, Japan) and penicillin/streptomycin (100 IU/50 μg/mL) (Invitrogen Japan) at 37° C in a humidified atmosphere containing 5% CO_2. Phase-contrast images were acquired using a microscope (IX51, Olympus, Tokyo, Japan) equipped with a digital camera (DS-US, Nikon, Tokyo, Japan).

Immunofluorescence staining

HSC-4 and HSC-3 cells were cultured for 48 hours on 0.1% gelatin-coated glass-coverslips and fixed in 4% paraformaldehyde in phosphate-buffered saline (PBS) for 30 minutes, permeabilized for 10 minutes with 0.1% Triton X-100 in PBS, and blocked with 3% bovine serum albumin (Nacalai Tesque, Kyoto, Japan) in PBS for 1 hour at room temperature. These cells were stained with primary antibodies against E-cadherin (1:200; #3195, Cell Signaling Technology Japan, Tokyo, Japan), N-cadherin (1:200; #13116, Cell Signaling Technology Japan), Vimentin (1:200; #5741, Cell Signaling Technology Japan), SLUG (1:200; #9585, Cell Signaling Technology Japan), SNAIL (1:200; #3879, Cell Signaling Technology Japan), and ZO-1 (clone T8-754, kindly provided by Dr. Masahiko Ito, Dokkyo Medical University, Japan and Dr. Mikio Furuse, National Institute for Physiological Sciences, Japan) for 3 hours at room temperature, and the following secondary antibodies were used: Alexa Fluor488-conjugated donkey anti-rabbit IgG (1:500; Invitrogen Japan) or Cy3-conjugated donkey anti-mouse IgG (1:500; Jackson Laboratory, ME, USA) for 1 hour at room temperature. Control rabbit and mouse IgG were purchased from Chemicom International, Inc. (CA, USA) and Upstate Biotechnology (NY, USA), respectively. DAPI (0.1 μg/ml; 4′,6-diamidino-2-phenylindole; Invitrogen Japan) counterstaining and Alexa Fluor633-conjugated Phalloidin (1:2,500: Invitrogen, Japan) staining were performed. Stained specimens were examined using a fluorescence microscope (BZ-X710).

Western blot analysis

HSC-4 and HSC-3 cells were cultured in the presence or absence of ANA-12, a TRKB inhibitor (1, 5 μM), and then lysed in SDS-PAGE sample buffer. These lysates were aliquoted and stored at −80° C until used. Protein concentrations were determined using Coomassie brilliant blue-staining protein spot for each specimen, with a standard concentration of protein. Equivalent amounts (2 or 5 μg) of protein were loaded for each condition and resolved by SDS-PAGE, electrophoretically transferred to PVDF membrane (Merck Millipore, Darmstadt, Germany), blocked with 5% BSA in Tris-buffered saline (TBS), and probed overnight at 4° C with antibodies against E-cadherin (1:1,000), N-cadherin (1:1,000), VIMENTIN (1:1,000), SLUG (1:1,000), SNAIL (1:1,000), and β-Actin (1:20,000; clone AC-15, Sigma-Aldrich Japan, Tokyo, Japan). Blots were then washed with TBS containing 0.05% Tween 20 (TBST), incubated for 1 hour with horse radish peroxidase (HRP)-conjugated anti-rabbit IgG or anti-mouse IgG antibodies (1:5,000; Jackson Laboratory) in TBST supplemented with 5% BSA at room

temperature, and subsequently washed with TBST. Blots were then developed using a Luminata Western HRP substrate (Merck Millipore). Signals were detected and documented with the densitometry system LAS3000 (Fujifilm, Tokyo, Japan).

Orthotopic transplantation mouse model of OSCC

BALB/cSlc-*nu/nu* female mice were purchased from Japan SLC (Shizuoka, Japan) and housed in a laminar air-flow cabinet, in a barrier facility, under specific pathogen-free conditions. For transplantation, HSC-4 or HSC-3 cells were dissociated with Trypsin (0.05%)/EDTA (0.02%) and single-cell suspensions were prepared in sterile PBS. The viability of the cells was checked before injection (>95%). Under 2,2,2-tribromoethanol (Sigma-Aldrich Japan) anesthesia, 8-week-old BALB/cSlc-*nu/nu* mice were injected submucosally with 1.75×10^5 HSC-4 or HSC-3 cells (30 μL) of into the left side of the tongue of each mouse using a Hamilton syringe fitted with a 26-gauge needle. A TRKB specific inhibitor, ANA-12 (Abcam, Cambridge, UK), was administered at 0.5 mg/kg into the mice by intraperitoneal injections 24 hours after the transplantation. The injections were performed every 12 hours for 20 days. All studies on animal models were approved by the Ethical Committee of the Osaka Medical College and performed according to its guidelines.

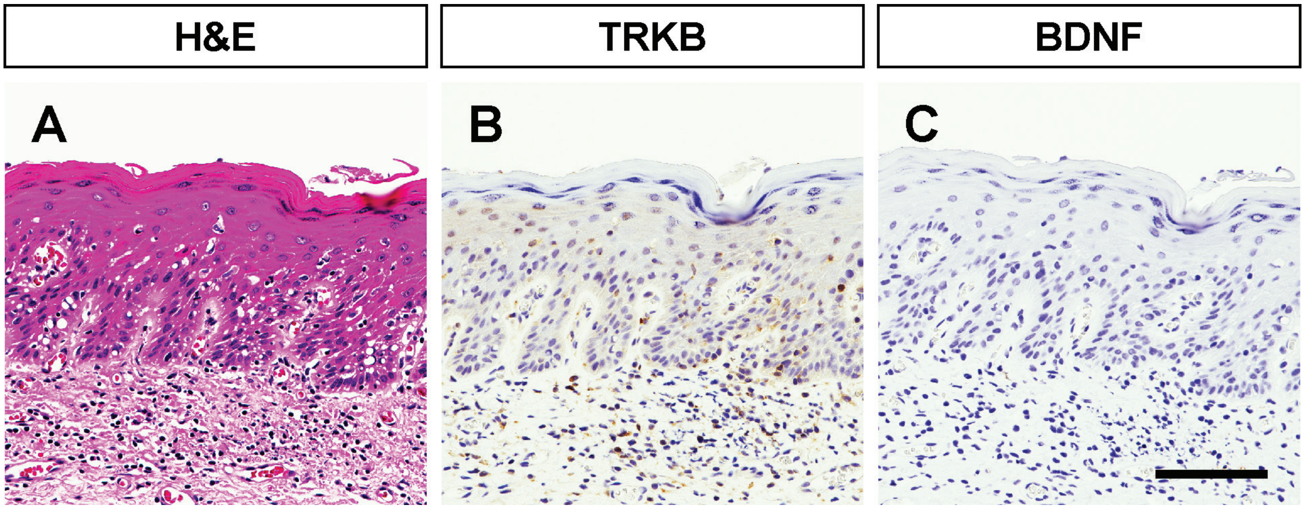

Supplementary Figure 1: TRKB and BDNF expressions in normal mucosa. Normal-appearing mucosae adjacent to OSCC in patients were stained with H&E (**A**), anti-TRKB antibody (**B**), and anti-BDNF antibody (**C**). Note, TRKB was weakly expressed in the stratum basal, which consists of proliferating cuboidal cells. Bar: 100 μm.

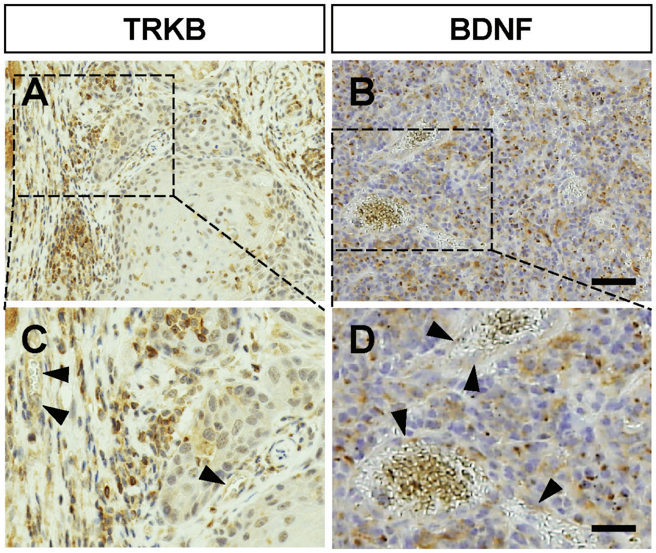

Supplementary Figure 2: Overexpression of TRKB and BDNF in vessels associated with OSCC tumors. Representative images of the immunohistochemical detection of TRKB and BDNF in tumor tissues obtained from patients with OSCC. Arrowheads show TRKB- or BDNF-overexpressing tumor-associated vessels. Bars represent 200 µm (**A** and **B**) and 400 µm (**C** and **D**).

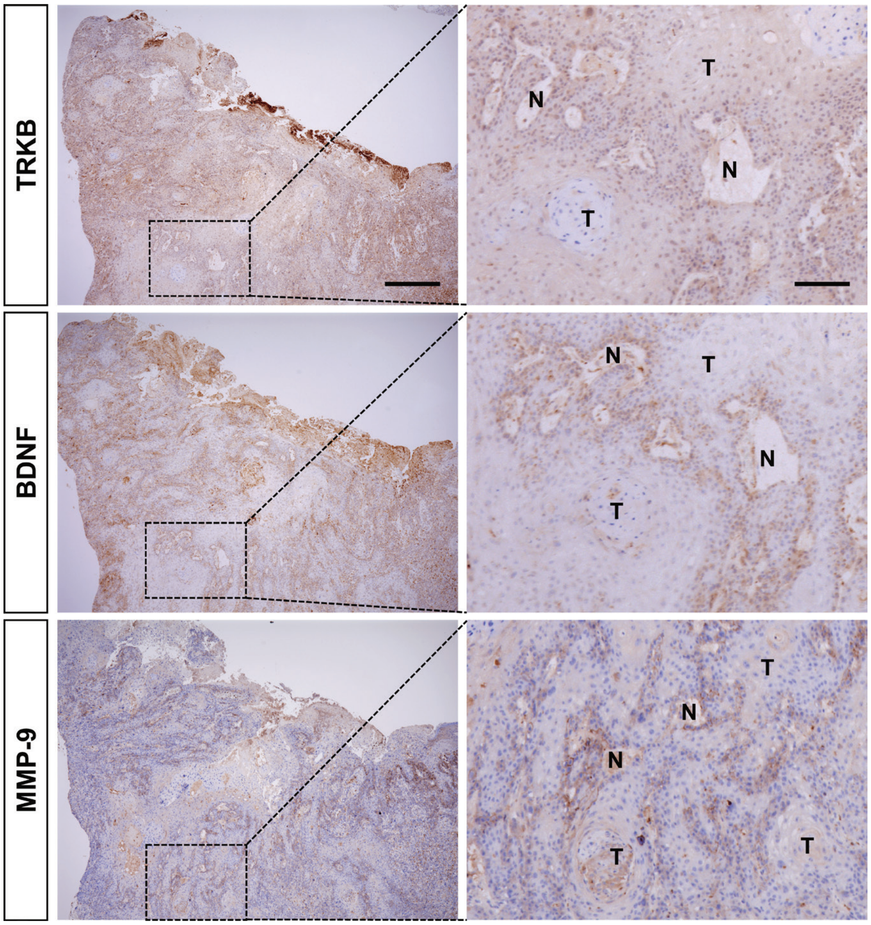

Supplementary Figure 3: Elevated expression of TRKB and BDNF in marginal areas of WD-OSCC tumors. Representative immunohistochemical images of TRKB and BDNF in tumor tissues from patients with WD-OSCC. The serial section images showed that the expression of TRKB and BDNF was increased in the marginal areas of the tumors visualized with MMP-9-staining. Tumoral (T) and non-tumoral (N) areas are indicated. Bars represent 500 µm (left panels) and 125 µm (right panels).

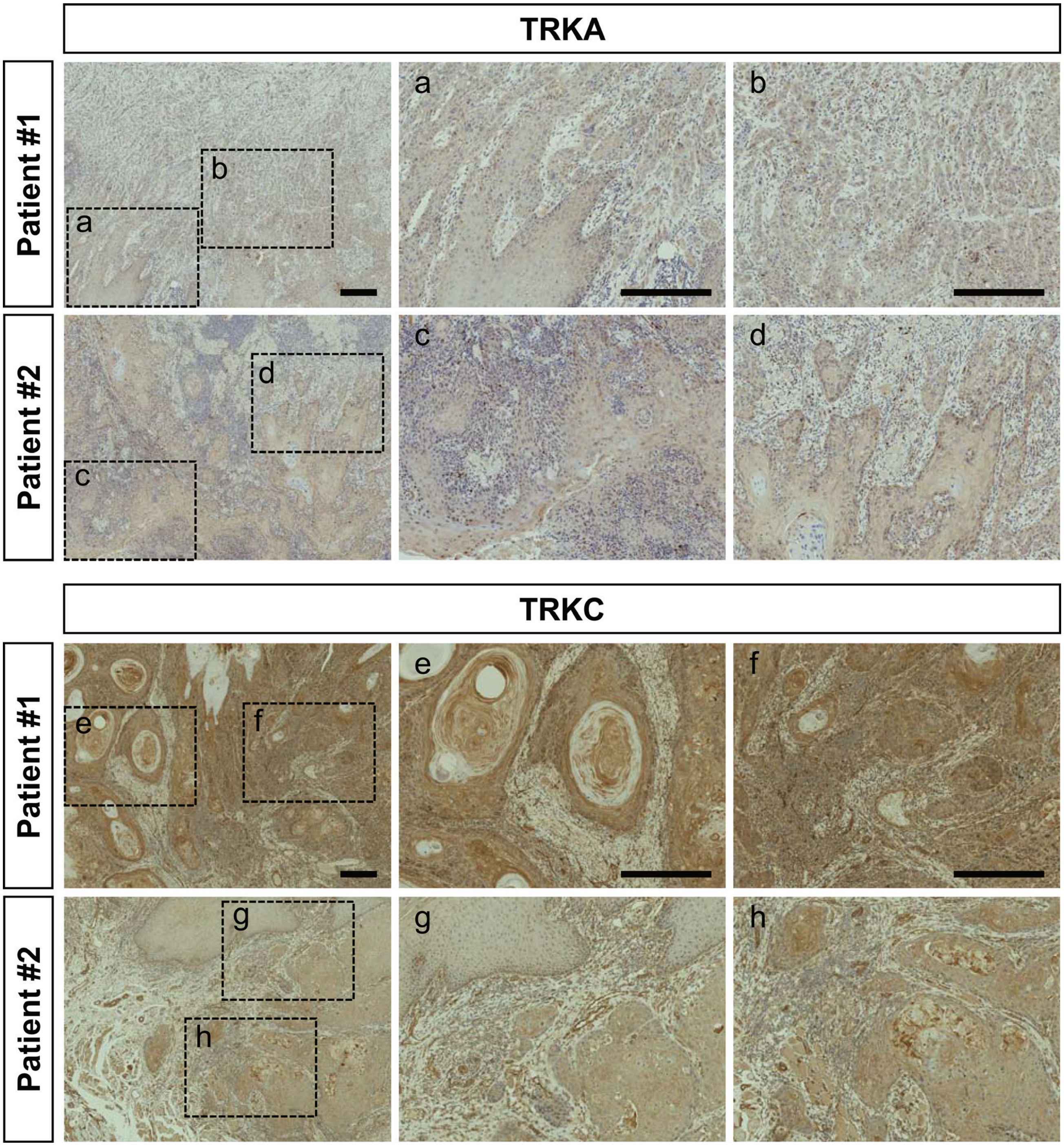

Supplementary Figure 4: Overexpression of TRKA and TRKC in OSCC tumors. Representative immunohistochemical images of TRKA and TRKC in tumor tissues from the different patients with WD-OSCC. They were overexpressed in the OSCC tumor cells and tend to be more highly expressed in the marginal area of WD-OSCC tumors and in MD/PD-OSCC tumors. However, unlike TRKB, their expression levels were not significantly different between in WD-OSCC tumors and in MD/PD-OSCC tumors. Note, in the some regions of the tumors, the expression levels in WD-OSCC tumor cells were as higher as in MD/PD-OSCC tumor cells. Bars: 200 µm.

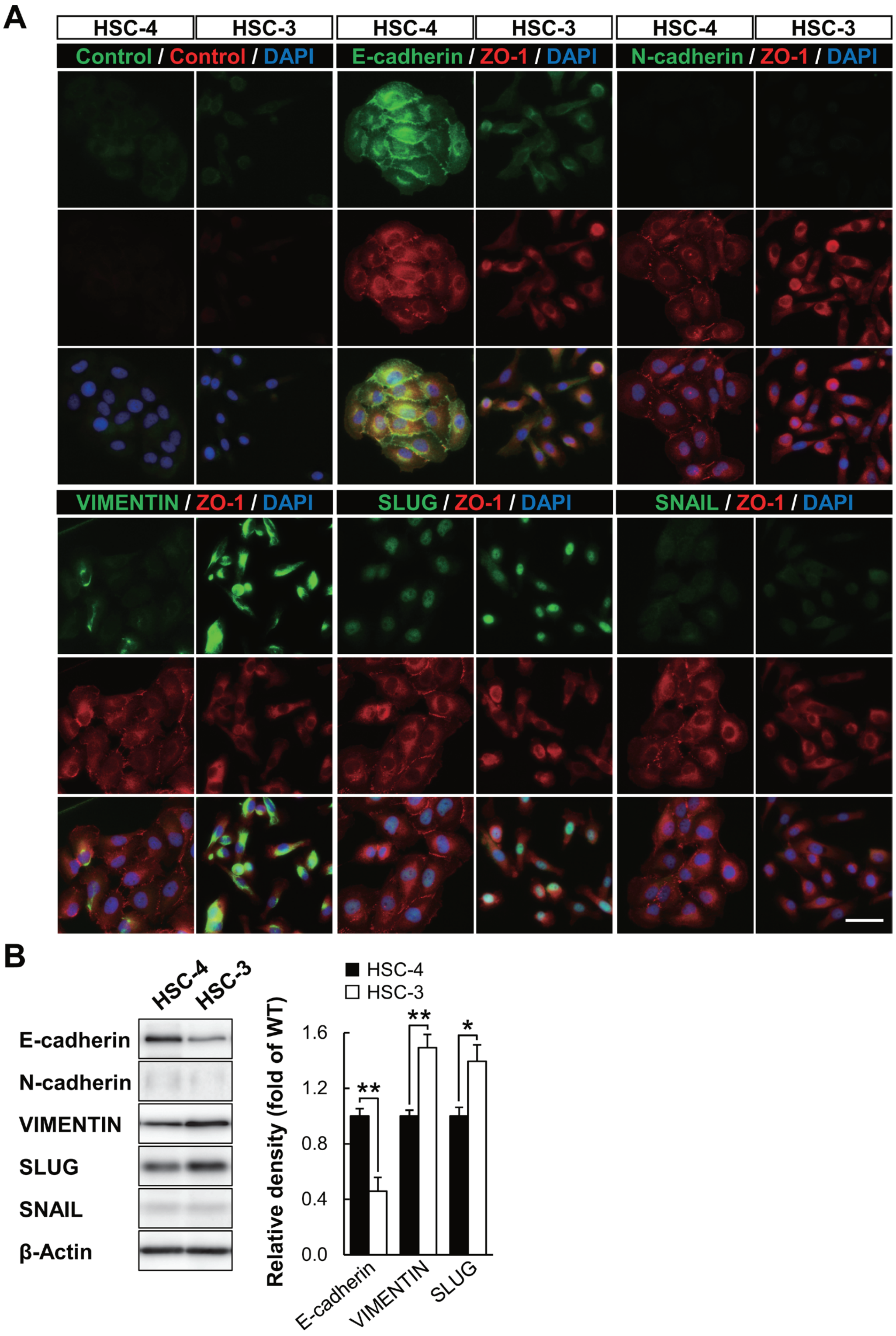

Supplementary Figure 5: Characterization of human OSCC cell lines, HSC-4 and HSC-3. Immunofluorescence (**A**) and Western blot analyses (**B**) of HSC-4 and HSC-3 cells were performed using antibodies against epithelial (E-cadherin) and mesenchymal (N-cadherin, VIMENTIN, SLUG, and SNAIL) markers. Bar: 50 μm. (**C**) Histograms show the means ± SEM (n = 3 in each cell line) of the densitometry analyses of the blot (HSC-4, filled bar; HSC-3, open bar) as a ratio against control value. β-Actin was used as the loading control. $^{*}P < 0.05$. Note: HSC-4 shows epithelial features, whereas HSC-3 cells show mesenchymal features.

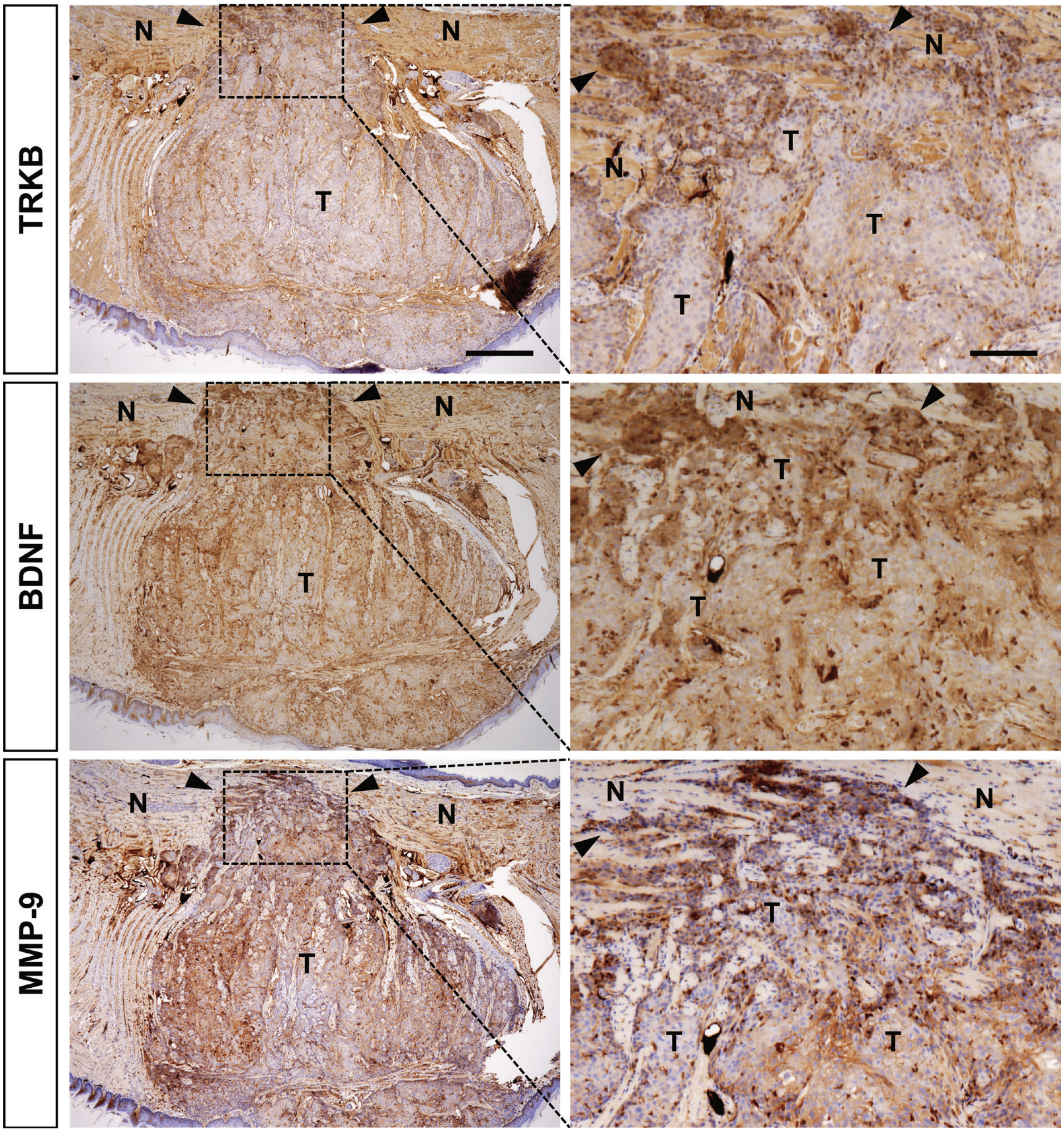

Supplementary Figure 6: Elevated expression of TRKB and BDNF in invasive front of OSCC tumors in mice. Representative immunohistochemical images of TRKB and BDNF in tumor tissues from mice transplanted with HSC-4 OSCC tumor cells. The serial section images showed that the expression of TRKB and BDNF was increased in the marginal area of the tumors visualized with MMP-9-staining. Arrowheads indicate the elevated expression of TRKB, BDNF, and MMP-9 at the invasive front of the tumors. Tumoral (T) and non-tumoral (N) areas are indicated. Bars represent 500 μm (left panels) and 125 μm (right panels).

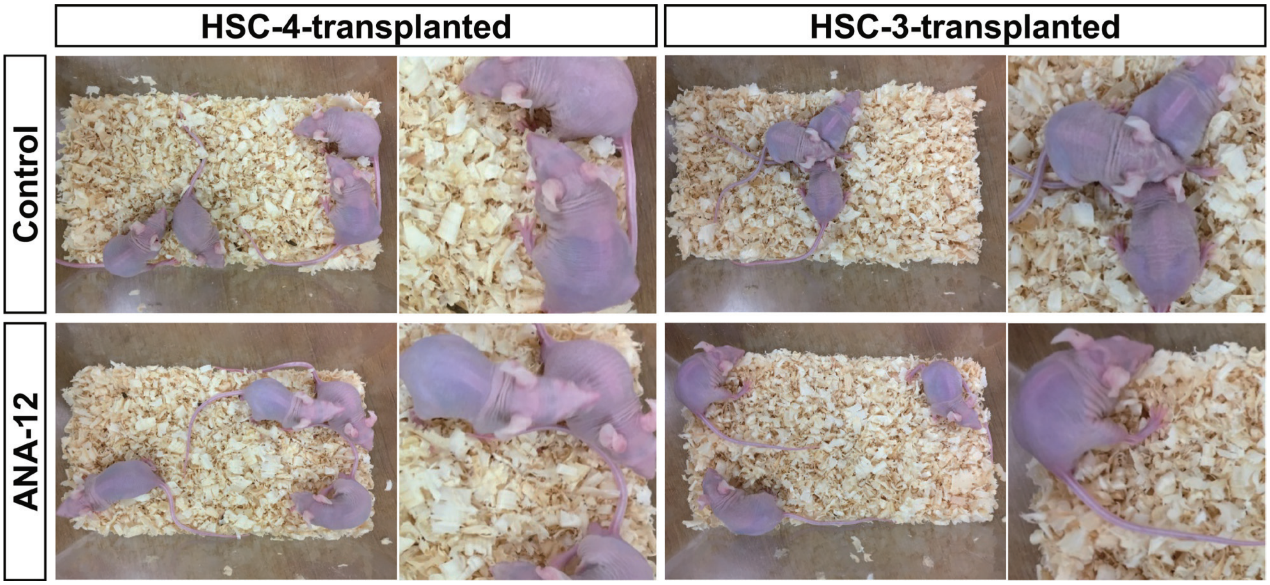

Supplementary Figure 7: HSC-4 and HSC-3 tumor-bearing mice administrated a TRKB-specific inhibitor. Eight-week-old BALB/cSlc-*nu/nu* female mice orthotopically transplanted with HSC-4 or HSC-3 tumor cells were intraperitoneally administrated control (DMSO) or ANA-12 (0.5 mg/kg, every 12 hours), and a TRKB-specific inhibitor, 24 hours after the transplantation. These mice were photographed 18 days after tumor cells transplantation.

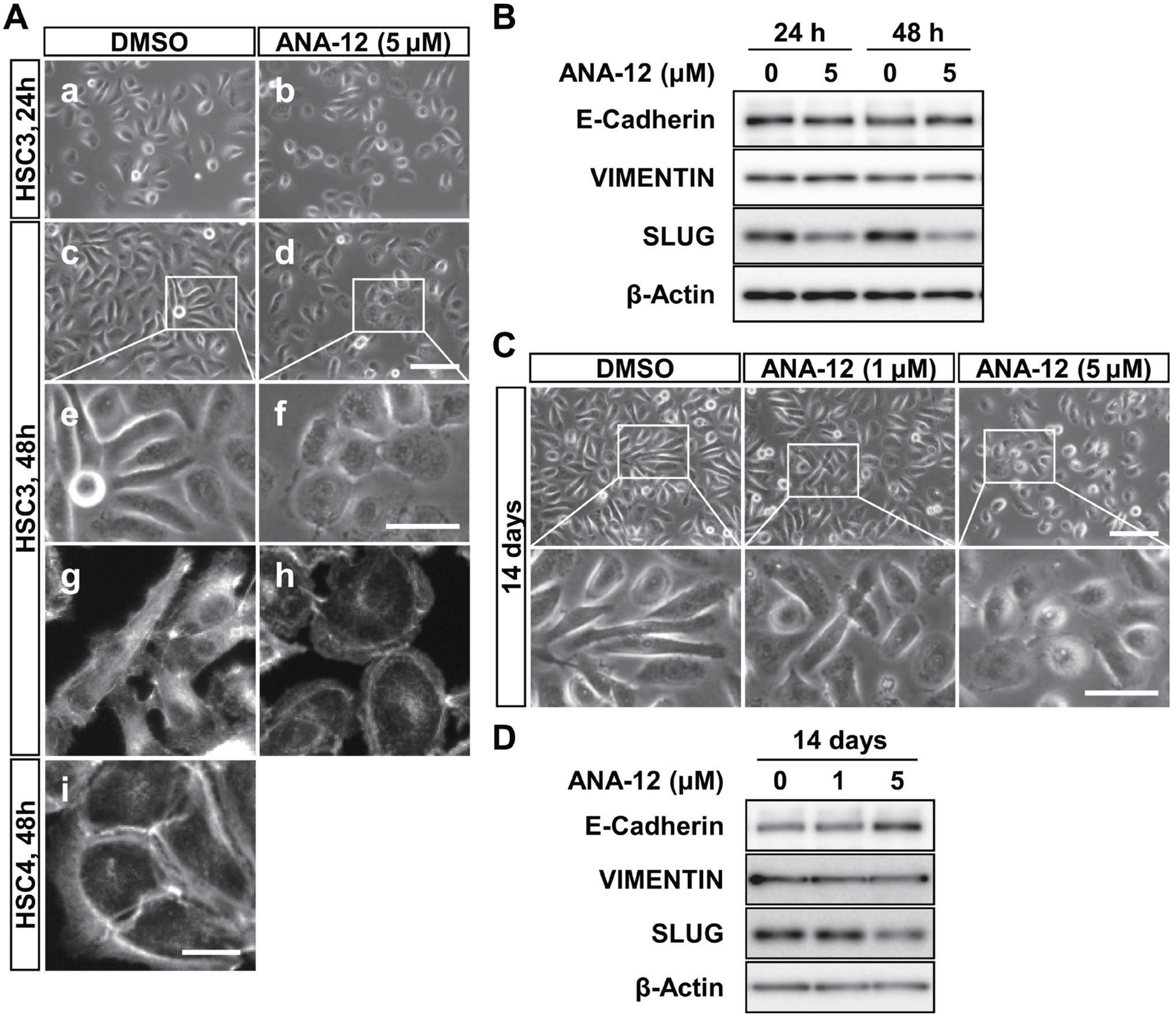

Supplementary Figure 8: Morphological change of HSC-3 cells by a TRKB-specific inhibitor. HSC-3 cells were cultured in the presence or absence of ANA-12 (5 μM) for the indicated times. (**A**) The effect of ANA-12 on cell morphology in HSC-3 cells was examined by a phase-contrast microscopy. Fluorescent staining with Alexa Fluor633-conjugated Phalloidin showed in panel g-i. Note, treatment of HSC-3 cells with ANA-12 caused a change in cell morphology from fibroblast-like, spindle-shaped form to epithelial-like form, as to HSC-4 cells. Bars: 100 μm (d), 50 μm (f), 200 μm (i). (**B**) The effect of ANA-12 on protein expression of E-cadherin, VIMENTIN, and SLUG was examined by Western blot analysis. β-Actin was used as the loading control. (**C** and **D**) The effect of long treatment of ANA-12 on cell morphology and protein expression in HSC-3 cells was examined by phase-contrast microscopy (C) and Western blot analysis (D). Bars: 100 μm (upper panel), 50 μm (lower panel).

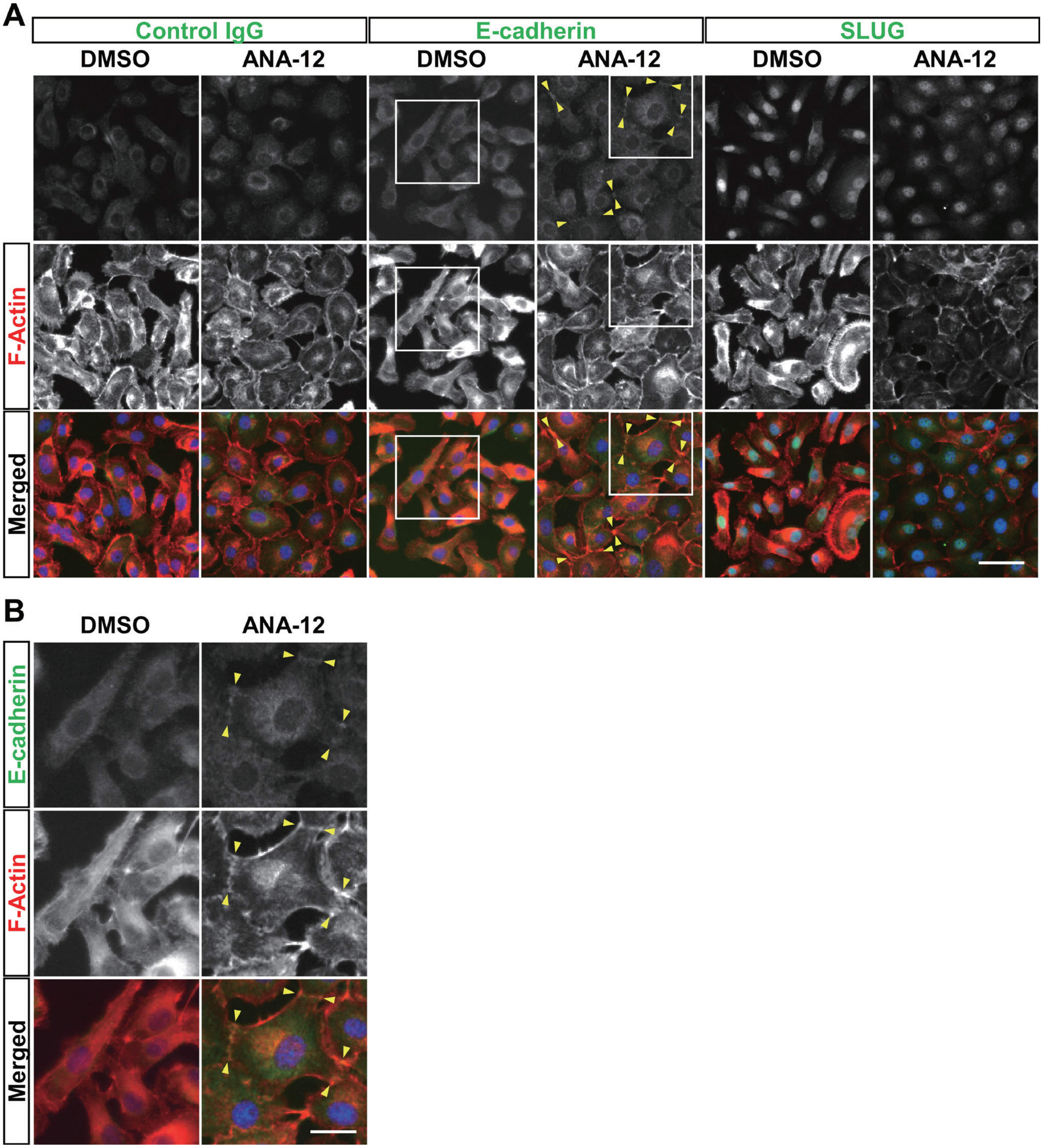

Supplementary Figure 9: Induction of mesenchymal-epithelial transition in HSC-3 cell by a TRKB-specific inhibitor. HSC-3 cells were cultured in the presence or absence of ANA-12 (5 μM) for 48 hours (**A** and **B**) and the effect of ANA-12 on protein expression and subcellular localization in HSC-3 cells was examined by a fluorescent microscopy using the indicating antibodies. Green, E-cadherin or SLUG; red, Phalloidin; blue, DAPI. Panels B shows the high magnification images of the insets in Panels A. Arrowheads indicate concentration of E-cadherin at cell-cell contact regions. Bars: 100 μm (A), 50 μm (B).
